# Mesothelin expression remodeled the immune-matrix tumor microenvironment predicting the risk of death in patients with malignant pleural mesothelioma

**DOI:** 10.3389/fimmu.2023.1268927

**Published:** 2023-10-12

**Authors:** Aline Nery Qualiotto, Camila Machado Baldavira, Marcelo Balancin, Alexandre Ab’Saber, Teresa Takagaki, Vera Luiza Capelozzi

**Affiliations:** ^1^ Laboratory of Genomic and Histomorphometry, Department of Pathology, University of São Paulo Medical School, São Paulo, Brazil; ^2^ Division of Pneumology, Heart Institute (Incor), Faculty of Medicine, University of São Paulo, São Paulo, Brazil

**Keywords:** malignant mesothelioma, mesothelin, PD-L1, immune cells, computational quantification, immunohistochemistry

## Abstract

**Background:**

The combination of immunobiological agents with immune checkpoint proteins is a promising treatment for malignant pleural mesothelioma (MPM). Mesothelin and anti-PD-L1 antibody-drug conjugates specifically target malignant neoplastic cells, inhibit the migration and invasion of neoplastic cells, and restore the immune landscape. In this study, we confirmed the importance of mesothelin and examined the relationship between mesothelin and the immune landscape of the tumor microenvironment (TME) in two MPM cohorts.

**Methods:**

The discovery cohort included 82 MPM cases. Tissue microarray slides were generated, and samples were processed for hematoxylin & eosin staining, immunohistochemistry, and immunofluorescence assays. The relationship between mesothelin, biomarkers of histogenesis, histological aggressiveness, PD-L1, immune cells (CD4, CD8, CD20, CD68), and collagen type I and type V fibers was evaluated by quantitative digital analyses. The outcome was the survival time until death from disease recurrence. The exploratory cohort included 87 malignant mesothelioma (MESO) patients from The Cancer Genome Atlas database.

**Results:**

Most patients were male (70.7%) with a history of asbestos exposure (53.7%) and with the epithelioid subtype (89%). Surgical resection was performed in 85.4% of patients, and 14.6% received chemotherapy; 59.8% of patients died from disease extension to the mediastinum. Low tumor mesothelin expression was associated with tumor necrosis and nuclear grade 1, whereas high mesothelin expression was significantly associated with the epithelioid histotype and high density of T cells CD8+, macrophages CD68+, and collagen type I fibers. Cox multivariate analysis showed a high risk of death for non-operated patients [hazard ratio (HR), 3.42 (1.15–10.16)] with low tumor mesothelin levels [HR, 2.58 (1.09–6.10)] and high PD-L1 and low infiltration of T cells CD4+ [HR, 3.81 (1.58–9.18)]. In the exploratory cohort, low mesothelin and high COL1A1 and COL5A1 expression were associated with poor overall survival.

**Conclusion:**

Tumor mesothelin expression associated with the TME immune landscape predicts the risk of death for patients with MPM and could be a new target for immunotherapy in MPM.

## 1 Introduction

Malignant pleural mesothelioma (MPM) is an aggressive malignancy primarily caused by asbestos exposure; it has a poor prognosis, with an overall survival (OS) of 6–13 months (1, 2). Despite established therapeutic guidelines, there are no effective treatments for this severe disease (3, 4). Patients with stage I and II disease receive multimodal treatment consisting of various cycles of chemotherapy, followed by surgery and/or radiotherapy. The standard chemotherapeutic approach is a combination of cisplatin or carboplatin with the antifolate pemetrexed (5, 6). However, the response rate to cisplatin treatment is approximately 14%, and the median OS of MPM patients treated with cisplatin is <7 months (7). The response to carboplatin is similar, with reported response rates of 6–16% (8). Further possible immunotherapy approaches ongoing phase I and II trials could be vaccines (e.g., CRS-207 and WT1 peptides) (9, 10) and gene modified lymphocytes, autologous T cells that, after gene transfer, express a chimeric antigen receptor (CAR) which enable the T cells to destroy target cells (11). Mesothelin-specific and fibroblast activation protein (FAP)-specific redirected T cells have shown *in vitro* and *in vivo* activity (12–15). However, effective biomarkers for predicting the response to treatment remain to be identified (16). These limitations highlight the need for further research to identify novel treatment strategies (1).

Exposure to asbestos fibers causes death of human mesothelial cells (HMs), a cell type that is particularly susceptible to asbestos fiber cytotoxicity (17). The combined effects of HMs and macrophages and the biopersistence of many mineral fibers allow some HMs to avoid cell death and undergo oncogenic transformation (18). The association of MPM with asbestos-induced carcinogenesis and the identification of the genes involved, such as mesothelin (MSLN), are promising findings (19). The human MSLN gene encodes a precursor protein of 622 amino acids that is processed by removal of N-terminal residues and linkage of glycosylphosphatidyl-inositol (GPI), which mediates its attachment to the cell membrane. The precursor is cleaved into a soluble peptide called ‘‘megakaryocyte potentiating factor’’ (20) and a GPI-anchored membrane-bound glycoprotein (mature mesothelin, MSLN) (21, 22). Although the MSLN protein is expressed at low levels in HMs, it is overexpressed in asbestos-exposed subjects (23, 24) and aberrantly expressed in several solid tumors, including gastric, lung, pancreatic, and ovarian cancers as well as in mesothelioma (25).

Preclinical studies showed that MSLN is involved in cell proliferation, anoikis resistant and survival (1, 17, 18), and its downregulation promotes drug-induced apoptosis and chemosensitivity (19, 20). Further studies have shown that MSLN promotes tumor cell survival and proliferation through NF-κB pathway activation, resulting in an increase of interleukin-6 level (26). Recently, He and colleagues demonstrated that MSLN control tumorigenicity and metastatic potential through epithelial-to-mesenchymal transition and stem properties of mesothelioma cell lines (27). Interestingly, genetic knockdown of MSLN significantly reversed EMT and attenuated stem cell properties, in addition to inhibiting tumor growth and metastasis; ectopic overexpression of MSLN induced the malignant phenotype of non-tumoral cells, supporting its role as an oncogene (27). However, the tumor model in these studies does not resemble the immune-matrix tumor microenvironment (TME) of human mesothelioma. To study the immune-matrix response induced by MSLN, a clinical-relevant human model is needed.

In the present study, we evaluated a clinical mesothelioma cohort to study the association between MSLN and remodeling of the immune matrix of the tumor microenvironment (TME). We evaluated tumor portion (nuclear grade, necrosis, BAP-1, MSLN) and TME portion (CD4, CD8, CD20, CD68, elastic fibers collagen type I and type V fibers) by quantitative digital analyses. We showed here that MSLN reshapes the immune matrix of the tumor microenvironment, recruiting T cells CD8+ and macrophages CD68+, increasing the collagen type I protein, and contributes to the establishment of functional and mechanical anti-tumor barrier in MPM mitigating the risk of death. Our results coincided with increased expression of CD8A, CD8B, MS4A1, CD68, MSLN, COL1A1 genes in the exploratory cohort. We showed here that MSLN expression remodeled the immune matrix of tumor microenvironment mitigating the risk of death in patients with malignant pleural mesothelioma.

## 2 Methodology

### 2.1 Discovery cohort

A retrospective study was performed using surgical samples (N=68) and large biopsies (N=20) from patients with malignant mesothelioma (MESO) obtained in partnership with the Heart Institute of the Hospital das Clínicas (InCor, Laboratory of Pathological Anatomy), the Cancer Institute of São Paulo (ICESP), and the Hospital das Clínicas of the Faculty of Medicine of the University of São Paulo (HC-FMUSP). The study included patients in stage III/IV and chemo-naïve.

A total of 246 cases of MESO were identified, of which those diagnosed as benign mesothelioma and its variants (papillary, well-differentiated cystic) and cytological examinations (pleural effusion) were excluded. Cases with inadequate histological specimens and those submitted for external review were also excluded. Finally, 82 samples were included in the study.

Patient data including sex, age, history of exposure to asbestos, histology, location of the disease, treatments performed, and survival were collected. Data were obtained through the electronic data system available in REDcap, which is hosted at ICESP. The primary endpoint was OS, which was defined as the time from first contact until death due to complications of the disease.

The present study was performed in accordance with Good Clinical Practice guidelines and the principles of the Declaration of Helsinki. The Ethics Committees of all participating institutions approved this study under opinion number 2.394.571.

### 2.2 Construction of tissue microarray slides

Tissue microarray (TMA) slides were generated for the 82 cases using histological blocks. For this purpose, three of the most representative tumor areas from the central, intermediate, and peripheral portions of 1 mm in diameter were selected from the original slides and blocks by experienced pathologists in hematoxylin-eosin stained samples. The marked tissues were extracted and transferred to paraffin blocks using precision mechanized equipment (MTA1, Manual Tissue Arrayer, Beecher Instruments, USA). Each cylinder was positioned in the receptor block according to a previously prepared map, with 0.3 mm spacing between samples. For each case, three cylinders were generated containing the central, intermediate, and peripheral portions of the most representative tumor areas. Kidney tissue samples were used as controls in the first three positions of the first row of the TMA matrix and served for orientation and positioning in the microscopic analysis.

### 2.3 Immunohistochemistry and immunofluorescence evaluation

For immunohistochemistry (IHC) and immunofluorescence (IF) assays, whole tissue and TMA sections were first tested by immunostaining to ensure uniformity. TMA sections (N = 82) were stained with immunoperoxidase and antibodies against the following proteins: MSLN (1:150; Biocare), D2-40 (1:1000; Dako); WT-1 (pre-diluted; Cellmarque); BAP-1 (pre-diluted, Santa Cruz); CD4 (1: 50, Novocastra), CD8 (1:400; Dako); CD68 (1:5000; Dako); PD-L1 (pre-diluted; Roche); and elastin (1:100; Santa Cruz). After preparation, the slides were analyzed under light microscopy to confirm reactivity and then digitally scanned at 40× magnification using a Pannoramic 250 scanner (3DHistech, Budapest, Hungary).

For the IF assay, TMA sections (N = 82) were deparaffinized in xylene, hydrated in graded ethanol, and exposed to a solution of 0.3% hydrogen peroxide and formic acid to inhibit endogenous peroxidase activity. For antigen retrieval, sections were incubated with a citrate buffer solution at pH 9.0 and heated in a Pascal pressure cooker (125°C for 1 min). Nonspecific sites were blocked with 5% bovine serum albumin in phosphate buffered saline (PBS) for 30 min at room temperature. Samples were incubated overnight at 4°C with antibodies against collagen I (1:700; Rockland Inc.) and collagen V (1:1400; Rockland Inc.). TMA sections were then washed in PBS with 0.05% Tween 20 and incubated for 60 min at room temperature with Alexa 488-conjugated goat anti-mouse IgG (1:200, Invitrogen, Eugene, OR, USA) and Alexa 488-conjugated goat anti-rabbit IgG (1:200, Invitrogen). Negative and autofluorescence controls were generated by incubating sections with PBS and normal rabbit or mouse serum instead of the specific antibody. Nuclei were counterstained with 0.4 mM/mL of 4’,6-Diamidino-2-Phenylindol, Dichloride (DAPI; Molecular ProbesTM, Invitrogen) for 15 min at room temperature. Finally, the specimens were mounted in buffered glycerol and visualized under an IF microscope (OLYMPUS BX51).

### 2.4 Quantification through image analysis

To quantify the IHC expression of different markers, the digitally scanned TMA slides were analyzed using QuPath software (version 0.4.3; Centre for Cancer Research and Cell Biology, University of Edinburgh, Edinburgh, Scotland), an open-source image analysis software platform (28).

The QuPath software provides a simple, automated, semi-supervised method to quantify TMAs. First, the scanned TMA slides were subjected to a series of automated assessments including staining vector analysis, total tissue area detection, tumor separation from non-tumoral areas, and cell detection. In the next step, the positivity limit threshold for each marker was established by trial and error. Then, after adjustment (validation by an expert pathologist), it was applied to the complete array.

At the end of the analysis, the number of positive cells per mm^2^ of tissue and the percentage of positive cells in the analyzed area were obtained using the QuPath software. For subsequent analyses, low expression refers to a positive cell density that is equal to or lower than the median expression in the cohort, and high expression refers to a positive cell density that is higher than the median.

### 2.5 Validation cohort (*in silico* analysis)

UALCAN (http://ualcan.path.uab.edu/), a user-friendly platform, was used to collect and analyze data from TCGA (29, 30). The relative mRNA expression of CD4, CD8A, CD8B, MS4A1, CD68, CD274, MSLN, COL1A1, and COL5A1 was obtained from TCGA Mesothelioma Pan-Cancer Atlas database. The platform normalizes the mRNA expression of the analyzed genes to transcript per million reads. The association between expression profiles and clinicopathological characteristics such as histological type, sex, and pathological stage was obtained from the UALCAN platform, and comparisons were made using Student’s T-test.

The clinicopathological characteristics of patients and the mRNA expression levels of nine genes of interest and their possible mutations were obtained from the cBio Cancer Genomics portal (cBioPortal; https://www.cbioportal.org/) (31, 32). This information was collected exclusively from TCGA Mesothelioma Pan-Cancer Atlas database.

The UCSC Xena tool (http://xena.ucsc.edu/) (33) was used to assess the expression profiles of the nine genes of interest, collect additional information, and query the prognostic value of these genes. The expression of the genes was used by the UCSC Xena tool to divide the patients into two groups (low vs. high expression) and plotted the Kaplan-Meier curves. This process was performed automatically by the platform, as well as the calculation of the log-rank value and the P-value determination for each of the generated curves.

The functional interactions between the proteins evaluated (CD4, CD8A, CD8B, MS4A1, CD68, CD274, MSLN, COL1A1, and COL5A1) were explored using the STRING platform (34, 35). This tool allows mapping of the protein-protein interaction (PPI) network and provides information on the function and biological processes involved in the enrichment of the genes analyzed and signaling pathways involved. For the enrichment analysis, we considered the strength and the false discovery rate. The strength describes how large the enrichment effect is and represents the result of expression: Log10(observed/expected). The false discovery rate describes how significant the enrichment is, in this case, the database shows the p-values corrected for multiple testing within each category using the Benjamini–Hochberg procedure.

### 2.6 Statistical analysis

The associations between protein expression, immune cells, clinicopathological features, and histotypes were analyzed using non-parametric tests (given the non-normal distribution of our data). The Cox proportional hazards model was used to analyze the association between OS rate and other covariates. Any parameters considered clinically relevant or those with P ≤0.2 in the univariate analysis were considered for the multivariate analysis (36). Correlations between variables were analyzed by Spearman correlation. For all analyses and graphical presentations, we used IBM SPSS statistical software (version 22; Armonk, NY, USA) and RStudio (version 2022.02.0, PBC, Boston, MA, USA; https://www.rstudio.com). P <0.05 was considered significant.

## 3 Results

### 3.1 Discovery cohort

#### 3.1.1 Baseline characteristics

The demographic and clinicopathological characteristics of the 82 patients included in the study are shown in **Table 1**. In the discovery cohort, 70.7% of patients were male and 29.3% were female. All patients were diagnosed with MESO. The median age of the patients was 60 years (range, 35–91 years). Asbestos exposure occurred in 53.7% of the patients. Histologically, 89% were classified as epithelioid and 11% as sarcomatoid. The pleura was the primary site in 75.6% of MESO cases. The majority of patients (85.4%) had undergone surgical resection. All patients were in an advanced pathological stage (III/IV), and 14.6% had received chemotherapy. During the follow-up period, 59.8% of the patients died from disease complications. The median survival was 18.4 months (range, 0.23–46.4 months).

**Table 1:** Frequency of demographic and clinicopathologic characteristics of the patients (N=82).

Characteristics	Number (%) of patients
Age (years)^a^	
Median (range)	60 (35 – 91)
≤60	39 (47.6%)
>60	41 (50.0%)
Gender	
Male	58 (70.7%)
Female	24 (29.3%)
Asbestos Exposure	
Exposed	44 (53.7%)
Not exposed	38 (46.3%)
Histological subtypes	
Epithelioid	73 (89.0%)
Sarcomatoid	9 (11.0%)
Localization	
Pleural	62 (75.6%)
Extra Pleural	20 (24.4%)
Treatment	
Surgical resection	70 (85.4%)
Biopsy	12 (14.6%)
Adjuvant chemotherapy	
Chemotherapy	12 (14.6%)
Status^a^	
Alive	31 (37.8%)
Dead	49 (59.8%)
Survival	
Mean (range)	18.4 (0.23-46.4)

a Some cases had missing information: age (2); status (2).

**Figure 1:**
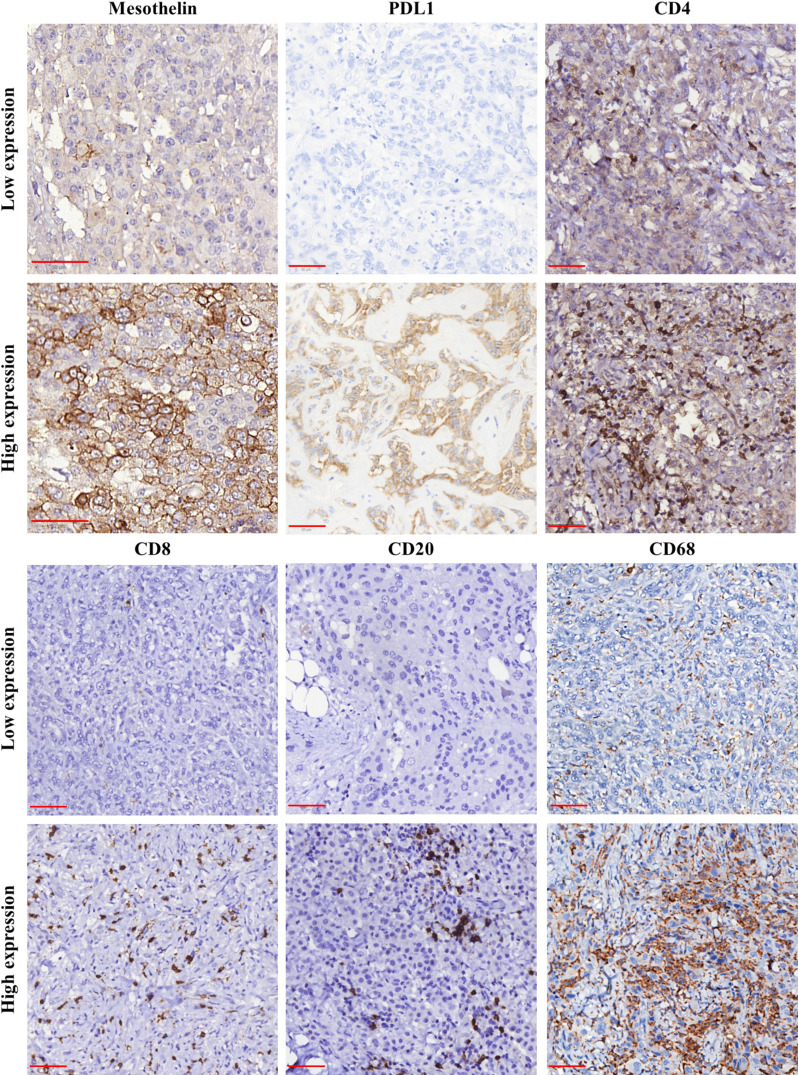
Immunohistochemical staining for mesothelin (MSLN), PD-L1, CD4, CD8, CD20, and CD68 in samples of malignant mesothelioma. The panel shows the differences between low (≤median) and high (>median) expression for each analyzed marker. Object-glass: 40×; scale-bar: 50 µm. Images captured using QuPath software.

**Figure 2:**
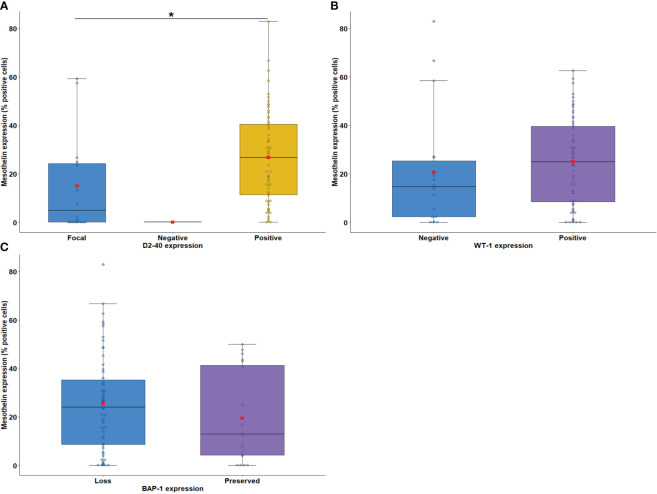
Boxplots showing the relationship between immunostaining for MSLN and D2-40 **(A)**, WT-1 **(B)**, and BAP-1 **(C)**. The solid bar represents the values of MSLN between the 25th and 75th percentiles; the black bar shows the median value; and the top and bottom brackets show the extreme values. D2-40, podoplanin; WT-1, Wilms Tumor Protein-1; BAP-1, BRCA1 associated protein-1 (ubiquitin carboxy-terminal hydrolase). Comparisons were performed using the Mann-Whitney and Kruskal-Wallis tests followed by Bonferroni’s multiple comparisons tests (P < 0.05). The square in red represents the mean expression value. *P < 0.05. Boxplots and comparison tests were generated by R studio software version 2022.12.0 + 353.

According to the median expression of MSLN, 21.05% of tumor cells were positive (interquartile range, 5.61–38.68% positive cells; **Figure 1**). As MSLN expression is also included for immunohistochemical diagnosis of MESO (34), our first step was to verify whether there was an association between the expression of MSLN and D2-40, WT-1, and BAP1. The results showed that high expression of MSLN by tumor cells was associated with diffuse expression of D2-40 (P = 0.009; **Figure 2A**). No difference was found between MSLN and WT1 (P = 0.14; **Figure 2B**), as well as MSLN and loss of BAP1 expression by tumor cells (P = 0.21; **Figure 2C**).

**Figure 3:**
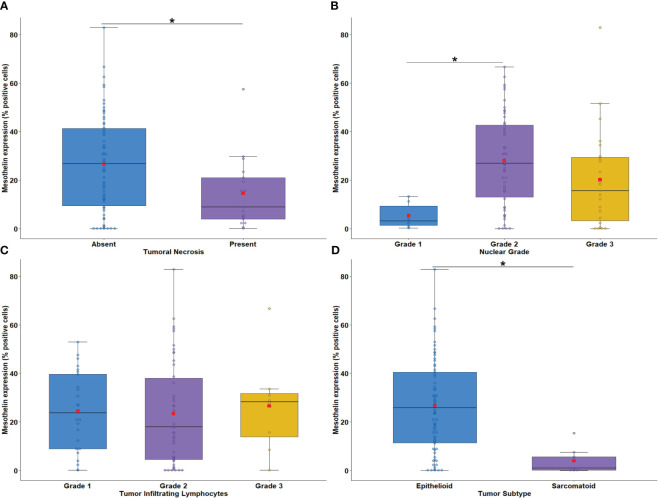
Boxplots showing the relationship between MSLN immunostaining and tumor necrosis **(A)**, nuclear grade **(B)**, intratumoral lymphocytes **(C)**, and malignant mesothelioma histotypes **(D)**. The solid bar represents the values of MSLN between the 25th and 75th percentiles; the black bar shows the median value; and the top and bottom brackets show the extreme values. Comparisons were performed using the Mann-Whitney and Kruskal-Wallis tests followed by Bonferroni’s multiple comparisons tests (P < 0.05). The square in red represents the mean expression value. *P < 0.05. Boxplots and comparison tests were generated by R studio software version 2022.12.0 + 353.

Next, we evaluated the association between tumor cell MSLN expression and histological biomarkers of tumor aggressiveness. Low MSLN expression was associated with tumor necrosis (**Figure 3A**; P = 0.03) and nuclear grade 1 (**Figure 3B**; P = 0.007). A similar expression of MSLN by tumor cells was observed according to tumor infiltrating lymphocyte (TIL) scores (**Figure 3C**; P = 0.70). Tumor cell MSLN expression was examined in epithelioid and sarcomatoid MESO histotypes, which showed that high expression of MSLN was significantly associated with MESO epithelioid cells, whereas low expression of MSLN was detected in sarcomatoid cells (**Figure 3D**; P < 0.001).

**Figure 4:**
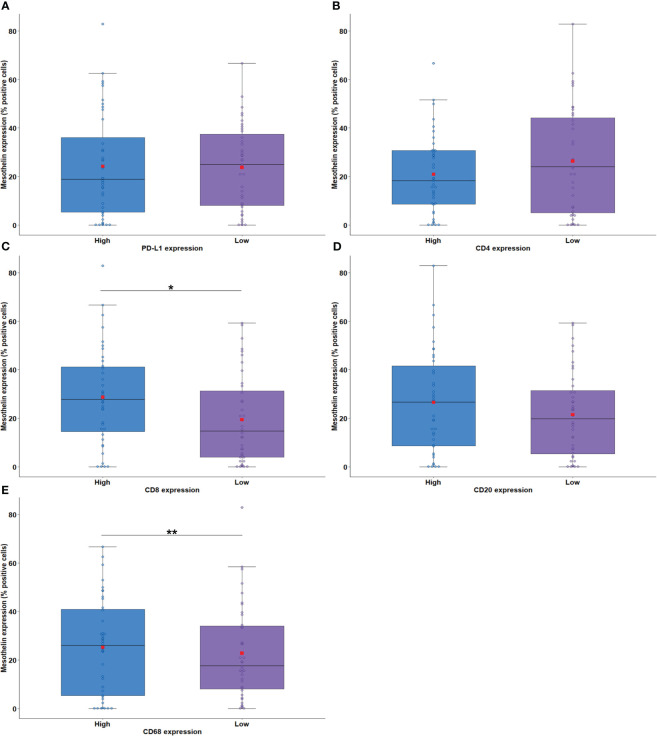
Boxplots showing the relationship between MSLN immunostaining and PD-L1 **(A)**, CD4 **(B)**, CD8 **(C)**, CD20 **(D)**, and CD68 **(E)**. The solid bar represents the values of MSLN between the 25th and 75th percentiles; the black bar shows the median value; and the top and bottom brackets show the extreme values. Comparisons between groups were performed using the Mann-Whitney test. The square in red represents the mean expression value. *P < 0.05; **P < 0.01. Boxplots and comparison tests were generated by R studio software version 2022.12.0 + 353.

Next, we examined the association of MSLN expression by tumor cells with PD-L1 expression and the density of immune cells in the tumor microenvironment (TME). The median expression of PD-L1 were just 0.58% positive cells (interquartile range, 0,18% –3.14% positive cells, **Figure 1**). There was no difference between the median expression of PD-L1 by tumor cells and the median expression of MSLN by tumor cells (**Figure 4A**; P = 0.79). Among the TILs present in the TME, the immune cell profile included T cells CD4+ (median, 44.97 positive cells/mm^2^; interquartile range, 18.17–204.49 positive cells/mm^2^; **Figure 1**), T cells CD8+ (median, 22.12 positive cells/mm^2^; interquartile range, 4,32 to 67.01 positive cells/mm^2^; **Figure 1**), B cells CD20+ (median, 9.74 positive cells/mm^2^; interquartile range, 1.83–41.82/mm^2^; **Figure 1**) and macrophages CD68+ (median, 1177.68 positive cells/mm^2^; interquartile range, 646.38–2030.77 positive cells/mm^2^; **Figure 1**). A high expression of MSLN by tumor cells was significantly associated with a high density of T cells CD8+ and macrophages CD68+ in the TME (**Figure 4C**; P = 0.01; and **Figure 4E**, P = 0.008, respectively). For T cells CD4+ and B cells CD20+ in the TME, a similar density was observed in both groups of MSLN expression (**Figure 4B**, P = 0.84; **Figure 4D**, P = 0.32).

**Figure 5:**
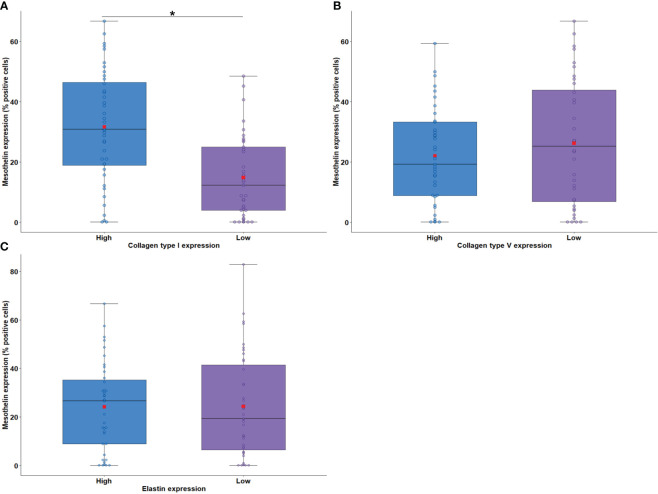
Boxplots showing MSLN expression in tumor cells associated with the density of collagen fiber type I **(A)** and type V **(B)** and elastin **(C)** in the tumor microenvironment. The solid bar represents the values of MSLN between the 25th and 75th percentiles; the black bar shows the median value; and the top and bottom brackets show the extreme values. Comparisons between groups were performed using the Mann-Whitney test. The square in red represents the mean expression value. *P < 0.05. Boxplots and comparison tests were generated by R studio software version 2022.12.0 + 353.

The relationship between MSLN expression by tumor cells and the density of elastic and collagen fibers in the TME was examined next. IF analysis detected collagen type I fibers (median, 0.26 positive fibers/mm^2^; interquartile range 0.66–1.12 positive fibers/mm^2^), collagen type V fibers (median, 2.71 positive fibers/mm^2^; interquartile range, 0.60–7.19 positive fibers/mm^2^), and elastin fibers (median, 3.58 positive fibers/mm^2^; interquartile range, 2.20–6.96 positive fibers/mm^2^) in the TME. A significant association was found between high MSLN expression by tumor cells and high density of collagen type I fibers in the TME (**Figure 5A**; P = 0.005). No difference was found between the median area occupied by collagen type V and elastin fibers in the TME and the median expression of MSLN by tumor cells (**Figure 5B**, P = 0.36; **Figure 5C**, P = 0.45).

#### 3.1.2 Patient characteristics and MSLN expression

The association between the characteristics of patients and MSLN expression is shown in **Table 2**. Low MSLN expression by tumor cells was detected in 20.3% of women and 32.9% of men, whereas high expression of MSLN by tumor cells was present in 39.2% of men and 7.6% of women (P = 0.04). Equally significant was the higher expression of MSLN in 46.8% of epithelioid tumor cells compared with 0.0% in the sarcomatoid histotype (P = 0.003). No more difference were found between MSLN expression and the other patient characteristics.

**Table 2:** Characteristics of the 82 patients with malignant mesothelioma by mesothelin expression levels (Fisher’s Exact Test; P<0.05).

	MSLN Expression ≤ 21.05%		MSLN Expression >21.05%		P value
	N	%	N	%	
**Age (median)**					0.82
≥ 61 yrs	20	26	19	24.7	
< 61 yrs	21	27.3	17	22.1	
**Sex**					**0.04**
Male	26	32.9	31	39.2	
Female	16	20.3	6	7.6	
**Topography**					0.08
Pleura	29	36.7	32	40.5	
Peritonium	9	11.4	5	6.3	
Testis	4	51.1	0	0.0	
**Asbestos**					1.73
No exposure	22	27.8	13	13.5	
Exposure	20	25.3	24	30.4	
**Histotypes**					**0.003**
Epithelioid	33	41.8	37	46.8	
Sarcomatoid	9	11.4	0	0.0	
**Treatment**					5.28
Biopsy	7	6.9	4	5.1	
Surgery	35	44.3	33	41.8	
Chemotherapy	8	10.1	4	5.1	
**Status**					0.34
Death	28	36.4	20	26.0	
Vital	13	16.9	16	20.9	
**Overall survival (mo)**					0.78
≤ 18.4	15	27.8	12	22.2	
> 18.4	13	24.1	14	25.9	

MSLN, mesothelin; yrs, years; mo, months.

Bolded values refer to a statistical significance of p-value (P<0.05).

#### 3.1.3 Correlation between MSLN, PD-L1, and TILs present in the TME

**Figure 6:**
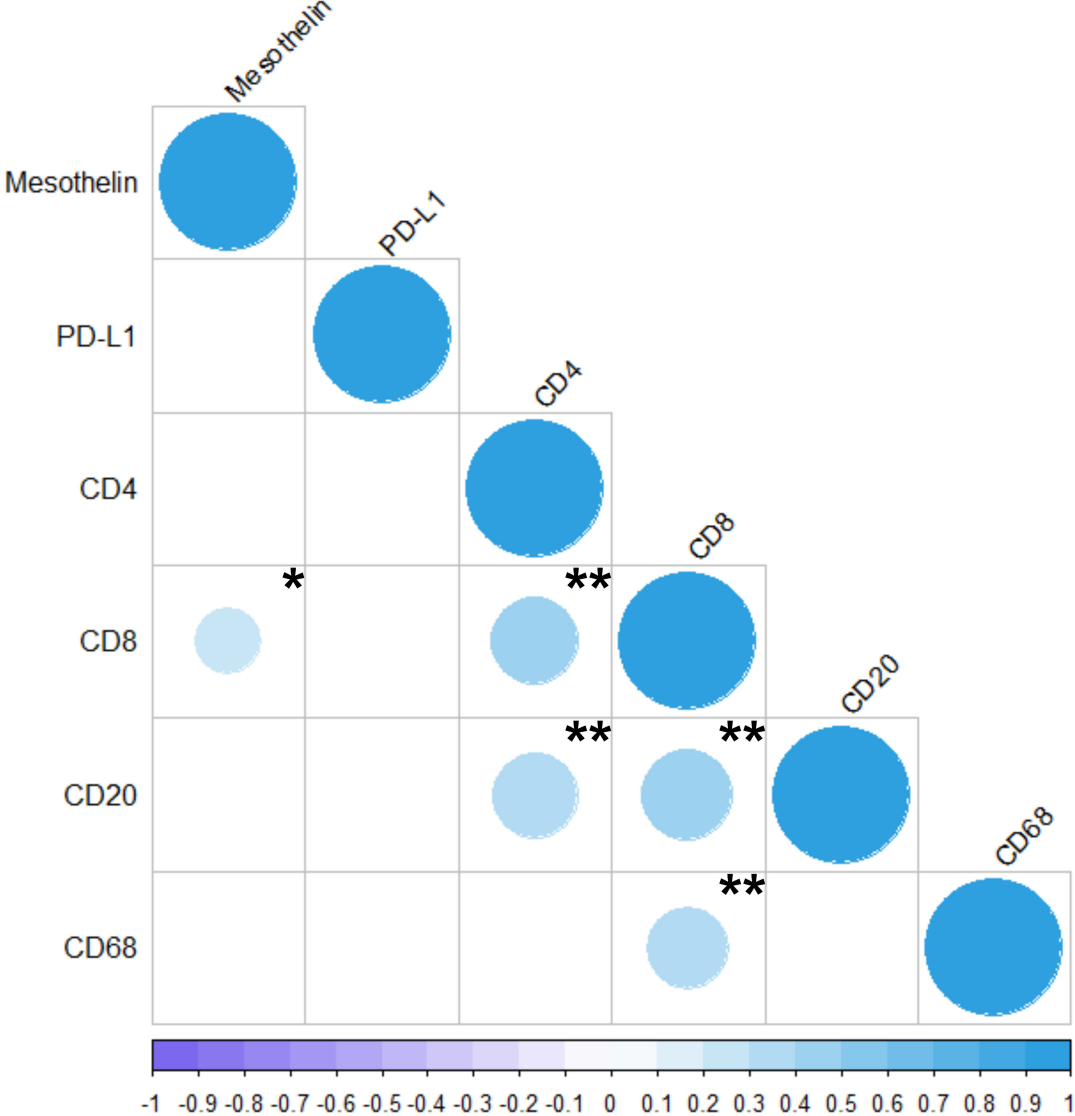
Correlation between the markers analyzed in the present study (MSLN, PD-L1, CD4, CD8, CD20, and CD68). The colors represents positive or negative correlations. The size of the dot represents Spearman’s rho; larger dots have values closer to |1|, indicating a stronger correlation. *P < 0.05; **P < 0.01. The Spearman Correlation Test and the Correlation Matrix were generated by R studio software version 2022.12.0 + 353.

The results of the Spearman correlation test are shown in **Figure 6**. Moderate correlations were observed between CD4 and CD8 (ρ = 0.418, P < 0.01), CD4 and CD20 (ρ = 0.391, P < 0.01), CD8 and CD20 (ρ = 0.439, P < 0.01), and between CD8 and CD68 (ρ = 0.370, P < 0.01). A weak correlation was observed between CD8 and mesothelin (ρ = 0.241, P < 0.03). PD-L1 was not directly correlated with any of the other variables.

#### 3.1.4 Survival analysis

The results of the Cox Regression analysis are presented in **Table 3**. First, univariate analysis was performed without adjustment to generate risk ratios with confidence intervals for each of the survival parameters. Considering a P value ≤0.2 for the variables in univariate analysis, a low hazard ratio (HR) was estimated for patients harboring epithelioid MESO [HR, 0.49 (0.20–1.18)], absence of necrosis [HR, 0.47 (0.23–0.96)], nuclear grade 2 [HR, 0.56 (0.28–1.14)], low tumor PD-L1 level [HR, 68 (0.35–1.30)], low TME occupied by collagen type I [HR, 0.83 (0.64–1.07)], and absence of chemotherapy [HR, 0.23 (0.11–0.49)]. By contrast, a high HR was determined for patients with low MSLN expression [HR, 1.73 (0.89–3.37)] and low infiltration of T cells CD4+ and T cells CD8+ in the TME [HR, 2.43 (1.23–4.80) and HR, 1.40 (0.72–2.67), respectively]. The effect of variables selected in the univariate analysis on OS was analyzed in a multivariate analysis. A poor OS was associated with non-operated patients [HR, 3.42 (1.15–10.16)] with low tumor MSLN expression [HR, 2.58 (1.09–6.10)], high levels of PD-L1, and low infiltration of T cells CD4+ in the TME [HR, 3.81 (1.58–9.18)].

**Table 3:** Variables associated with risk of death for patients with malignant mesothelioma using univariate and multivariate Cox regression.

	Univariate Analysis			Multivariate Analysis	
	HR (95% IC)^d^	HR ^c^	P value	HR (95% IC)	P value
Age (median)					
≤ 61 yrs	0.89 (0.46-1.71)	-0.12	0.72		
> 61 yrs (reference)					
Sex					
female	0.82 (0.40-1.66)	-0.20	0.57		
male (reference)					
Topography					
pleura	0.66 (0.19-2.22)	-0.42	0.50		
peritonium	0.71 (0.18-2.77)	-0.33	0.63		
testis (reference)			0.79		
Asbestos					
no exposure	0.70 (0.37-1.35)	-0.35	0.29	0.70 (0.29-1.67)	0.42
exposure (reference)					
Histotypes					
epithelioid	0.49 (0.20-1.18)	-0.71	0.11	0.92 (0.25-3.36)	0.90
sarcomatoid (reference)					
Necrosis					
no	0.47 (0.23-0.96)	-0.75	**0.04**	0.56 (0.23-1.33)	0.19
yes (reference)					
Nuclear grade (score)					
1	1.04 (0.39-3.23)	0.04	0.94	1.19 (0.33-4.32)	0.78
2	0.56 (0.28-1.14)	-0.57	0.11	0.68 (0.29-1.63)	0.39
3 (reference)			0.21		
Tumor infiltrating lymphocytes (score)					
1	0.60 (0.13-2.77)	-0.50	0.51		
2	0.69 (0.16-2.96)	-0.36	0.62		
3 (reference)			0.80		
Mesothelin (median/mm^2^)					
< 21.05	1.73 (0.89-3.37)	0.55	0.10	2.58 (1.09-6.10)	**0.03**
≥ 21.05 (reference)					
PD-L1 (median/mm^2^)					
< 0.35	0.68 (0.35-1.30)	-0.38	0.19	0.43 (0.19-0.94)	**0.03**
≥ 0.35 (reference)					
Immune cells (median/mm^2^)					
T cells CD4+					
< 44.97	2.43 (1.23-4.80)	0.89	**0.01**	3.81 (1.58-9.18)	**0.003**
≥ 44.97 (reference)					
T cells CD8+					
< 22.12	1.40 (0.72-2.67)	0.33	0.12	0.45 (0.18-1,10)	0.08
≥ 22.12 (reference)					
B cells CD20+					
< 9.74	1.01 (0.52-1.95)	0.01	0.97		
≥ 9.74					
Macrophages CD68+					
< 1177.78	1.09 (0.56-2.12)	0.09	0.78		
≥ 1177.78					
Collagen/elastin (median/mm^2^)					
***Collagen type I***					
< 0.26	0.83 (0.64-1.07)	-0.19	0.14	0.93 (0.41-2.08)	0.85
≥ 0.26					
Collagen type V					
< 2.71	1.22 (0.62-2.39)	0.20	0.56		
≥ 2.71 (reference)					
Elastin					
< 3.58	0.98 (0.92-1.04)	-0.01	0.63		
≥ 3.58 (reference)					
Surgery					
no	3.08 (1.33-7.13)	1.12	**0.009**	3.42 (1.15-10.16)	**0.02**
yes (reference)					
Chemotherapy					
no	0.23 (0.11-0.49)	-1.45	**0.0001**	0.44 (0.17-1,10)	0.08
yes (reference)					

a Univariate Analysis was performed without any adjustment to generate risk ratios with confidence intervals for individual risk for each of the survival parameters; ^b^Multivariate analysis was performed to analyze the effects of various risk parameters on survival;^c^HR, hazard risk (β coefficient);^d^CI, confidence interval. Chi square 32.80, P = 0.0001.

Bolded values refer to a statistical significance of p-value (P<0.05).

**Figure 7:**
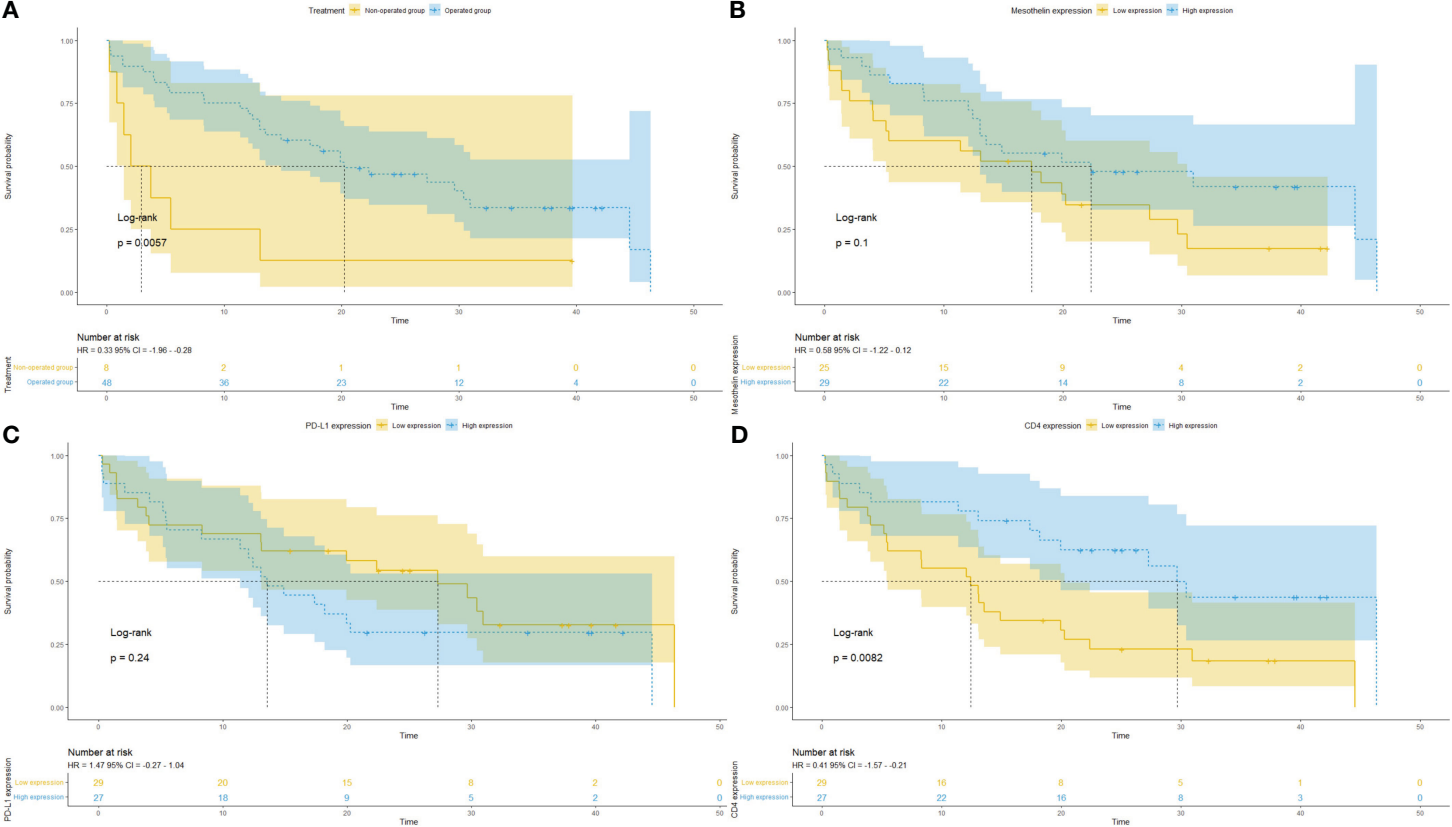
Kaplan–Meier curves of the probability of survival according to follow-up time in months in patients with malignant mesothelioma. Stratified curves according to **(A)** treatment (surgery vs. biopsy), **(B)** MSLN expression, **(C)** PD-L1 expression, and **(D)** CD4+ T cells.

The Kaplan–Meier plots of survival probability according to follow-up time in months in operated patients with positive tumor MSLN and PD-L1 expression and infiltration of T cells CD4+ are presented in **Figure 7**. Patients were divided into two groups with distinctly different average survival times. The top curve represents patients who underwent surgery, and the bottom curve corresponds to the non-operated group (**Figure 7A**). After dividing patients according to tumor MSLN expression, those with >21.05% positive cells/mm^2^ are grouped in the top curve and those with ≤21.05% positive cells/mm^2^ are at the bottom curve (**Figure 7B**). The groups with mean PD-L1 ≤0.58% positive cells/mm^2^ and T cells CD4+ >44.97 positive cells/mm^2^ are shown in the top curves, whereas those with PD-L1 >0.58% positive cells/mm^2^ and T cells CD4+ ≤44.97 positive cells/mm^2^ are shown in the bottom curves (**Figures 7C, D**).

### 3.2 Exploratory cohort

Transcriptome expression profiles and clinicopathological data were downloaded from TCGA database and included 87 MESO tumors. The expression levels of nine candidate genes encoding the proteins evaluated in the discovery cohort were examined in a validation cohort using TCGA data. **Supplementary Figure 1** shows the expression levels of the nine genes, namely, CD4, CD8A, CD8B, MS4A1, CD68, CD274, MSLN, COL1A1, and COL5A1. Mutations in CD4, MS4A1, CD274, and COL1A1 were detected in at least one mesothelioma sample (**Supplementary Figure 2**). Amplification of the CD4 gene was detected in one sample, and MS4A1 gene amplification was detected in two samples. In the CD274 gene, the L224F missense mutation was observed in one sample and amplification (driver) was observed in other samples. Amplification of the COL1A1 gene was detected in four mesothelioma samples.

#### 3.2.1 Clinical impact of genes evaluated by TCGA database analysis

The clinical impact of CD4, CD8A, CD8B, MS4A1, CD68, CD274, and COL1A1 was determined using TCGA database analysis. The correlation between differentially expressed genes and clinicopathologic characteristics and survival was examined in 87 patients with a median age of 64 years who were predominantly male (81.6%). This independent cohort was used to confirm the clinical impact of the nine genes in MESO. As shown in **Supplementary Figure 3**, CD4, CD8A, CD8B, MS4A1, CD68, and CD274 were upregulated in sarcomatoid MESO compared with other histotypes. Significantly high gene expression levels were observed for MSLN in epithelioid MPM (P < 0.01) and COL5A1 in biphasic MESO (P < 0.05).

CD8A, CD8B, and CD274 expression was significantly higher in men than in women with MESO (P < 0.05, P < 0.05, and P < 0.01, respectively; **Supplementary Figure 4**). High expression levels of CD4 were significantly associated with stage IV compared with stage I (P < 0.05), whereas high expression of MSLN was more frequent in stage II than in stage I (P < 0.05) and stage III (P < 0.05) patients, with significant differences between stages I and II (P < 0.05) and stages II and III (P < 0.05) (**Supplementary Figure 5**).

The correlations among the expression levels of the nine genes in TCGA database were also explored (**Supplementary Figure 6**). A strong direct correlation was found between CD4 and CD68 (ρ = 0.73, P < 0.001), CD8A and CD8B (ρ = 0.92, P < 0.001), and COL1A1 and COL5A1 (ρ = 0.89, P < 0.001). A moderate correlation was observed between CD4 and CD8A (ρ = 0.54, P < 0.001), CD4 and CD8B (ρ = 0.54, P < 0.001), CD8A and MS4A1 (ρ = 0.40, P < 0.001), CD8A and CD68 (ρ = 0.45, P < 0.001), CD8A and CD274 (ρ = 0.46, P < 0.001), CD8B and MS4A1 (ρ = 0.34, P = 0.01), CD8B and CD68 (ρ = 0.49, P < 0.001), and between CD8B and CD274 (ρ = 0.38, P < 0.001). An inverse moderate correlation was observed between MSLN and COL1A1 (ρ = -0.43, P < 0.001) and MSLN and COL5A1 (ρ = -0.54, P < 0.001).

These results indicate that the expression levels of these genes could be used for classifying MESO molecular subtypes.

#### 3.2.2 MSLN, COL1A1, and COL5A1 as independent survival biomarkers

The association between the expression levels of nine genes, namely, CD4, CD8A, CD8B, MS4A1, CD68, CD274, MSLN, COL1A1, and COL5A1 and survival was evaluated in patients with MESO. Kaplan–Meier analysis showed that low MSLN and high COL1A1 and COL5A1 expression levels were significantly associated with poor OS (MSLN, P = 0.002; COL1A1, P = 0.007; and COL5A1, P< 0.001; **Supplementary Figure 7**).

#### 3.2.3 Functional enrichment analysis

The molecular organization of the PPI network is shown in **Supplementary Figure 8**. The network is composed of differentially connected nodes; each node represents a protein, and the edges represent their dynamic interactions, thus providing a P-value for the PPI enrichment (P = 3.23e-10). This value indicates that, as a group, these proteins are at least partially biologically connected.

#### 3.2.4 Putative biological functions determined by pathway enrichment analysis

Gene Ontology analysis was performed to identify the biological processes and molecular functions involving these nine proteins. For “biological processes”, the strongest associations were “collagen biosynthetic process”, “adaptative immune response”, and “lymphocyte activation” (**Supplementary Table 1**). For “molecular functions”, the main results were “platelet-derived growth factor binding”, “MHC class I protein binding”, and “MHC protein binding” (**Supplementary Table 1**).

KEGG pathway analysis results showed that the main pathways related to the genes identified were “primary immunodeficiency”, “antigen processing and presentation”, and “hematopoietic cell lineage” (**Supplementary Table 1**). In “Reactome Pathways” the main results for the nine proteins were “PD-1 signaling”, “Nef-mediates down-modulation of cell surface receptors by recruiting them to clathrin adaptors” and “sydecan interactions” (**Supplementary Table 1**).

## 4 Discussion

In this study, analysis of the baseline characteristics of the discovery cohort confirmed the need to identify new predictive and prognostic biomarkers in MPM. Consistent with previous reports (37, 38), MPM was more common in men than in women, frequently associated with asbestos exposure, and most tumors were in stages III/IV with a poor response to surgical resection and chemotherapy. During the follow-up period, approximately 60% of patients died from expansion of the disease to the mediastinum. The median OS was 18.4 months. Although several therapeutic agents have been tested for the treatment of MPM (39) including immune checkpoint inhibitors (40), the treatment options for this disease remain limited. The proportion of MPM patients who achieve a prolonged response and survival remains low, and new therapeutic strategies are needed. A potential therapeutic assay using monoclonal antibodies targeting different MSLN epitopes covering the proximal membrane-regions could result in effective antibody-dependent cell-mediated cytotoxicity (25). These data prompted us to perform a comprehensive analysis of MSLN and its relationship with patient outcome, as well as the relationship between MSLN and the immune landscape in 82 cases of MESO.

The recent phase III trial Checkmate-743 revealed that the combination of ipilimumab and nivolumab presented a statistically significant improvement on overall survival compared with pemetrexed and platinum chemotherapy (median overall survival (OS) of 18.1 versus 14.1 months, p = 0.002), especially for non-epithelioid subtypes (41). Moreover, the 2/3 Keynote-483 trial (NCT02784171) combining pembrolizumab and chemotherapy improved overall survival (OS) vs chemotherapy alone, in patients with unresectable advanced or metastatic malignant pleural mesothelioma (42). However, the proportion of MPM patients who achieve a prolonged response and survival remains low, and new therapeutic strategies are needed. Because of its prevalence in cancers, MSLN has recently been targeted for immunotherapy (7), while the soluble MSLN fragment has been investigated as a biomarker for cancer diagnosis (8). In addition, soluble MSLN is a specific biomarker of MPM in asbestos-exposed subjects (43), and the diagnostic and prognostic value of MSLN in early MPM was investigated previously (19, 44, 45). A potential therapeutic assay using monoclonal antibodies targeting different MSLN epitopes covering the proximal membrane regions could result in effective antibody-dependent cell-mediated cytotoxicity (46). Among them, the clinical trials with SS1P, an immunotoxin that contained a murine anti-mesothelin antibody and a portion of Pseudomonas exotoxin, showed its clinical effectivity hampered due to the occurrence of neutralizing anti-drug antibodies (ADAs) (47, 48). Another trial assessed the combination of SS1P with pentostatin and cyclophosphamide, which showed an effective decrease in the development of ADAs tested on ten patients with chemotherapy-refractory mesothelioma (eight pleural and two peritoneal). Among these patients, three patients presented a durable PR (>14 months) and three others had SD (49). More recently, a trial with LMB-100 anti-mesothelin immunotoxin less immunogenic compared to SS1P, showed disappointing response rates, and a reduced clinical efficacy as a monotherapy (50). There are many other ongoing trials targeting a single factor to effectively manage MPM, instead of designing combination strategies that work synergistically at multiple levels.

Soluble MSLN is a specific biomarker of MPM in asbestos-exposed subjects (43), and the diagnostic and prognostic value of MSLN in MPM was investigated previously (19). In this study, we found that MSLN was expressed in >64% of MPM tumor specimens (49% showing high expression), suggesting that high MSLN expression may predict the response to treatment and patient outcomes. As expected, high tumor cell MSLN expression coincided with diffuse D2-40 expression by mesothelioma cells because both proteins are located in the cellular membrane, while nuclear expression of WT-1 and BAP-1 showed non-specificity with MSLN. Conversely, low expression of MSLN by tumor cells was associated with tumor necrosis and nuclear grade. This was consistent with Bharadwaj et al. (51), who reported that high MSLN expression increases resistance to tumor necrosis factor-α–induced apoptosis through the activation of Akt/phosphoinositide 3-kinase/nuclear factor κB signaling. Although MSLN was overexpressed in MESO, the present cohort showed inter-patient heterogeneity in MSLN expression. High MSLN levels were detected in 88% of the epithelioid MESO cohort. These data are consistent with the degree of MSLN-positivity observed in a comprehensive atlas of MSLN immunostaining (52).

In the discovery cohort, high expression levels of MSLN were associated with a high density of T cells CD8+, and macrophages CD68+. However, the expression of T cells CD4+ and B cells CD20+ in the TME does not present an association with MSLN expression. Studies suggest that MSLN is a strongly immunogenic protein. Thomas et al. reported that in patients with pancreatic adenocarcinoma, injection of granulocyte macrophage colony-stimulating factor-secreting pancreatic tumor cells induced a strong MSLN-specific CD8+ T-cell immune response (53). MSLN-specific IgG antibodies were identified in the serum of patients with advanced mesothelioma and ovarian cancer (54). These results indicate that MSLN-specific B-cell and T-cell responses against MSLN-expressing cancer cells contribute to the prolonged OS.

The association between increased MSLN expression in tumor cells and increased collagen type I fibers suggests that this protein could be involved in maintaining the plasticity of the TME to avoid invasion by malignant cells (55).

The present results suggest that PD-L1 and MSLN overexpression is associated with the mortality of MPM. We found that the risk of death was three-fold higher among non-operated patients with low tumor cell MSLN expression, high PD-L1 levels, and low infiltration of T cells CD4+ in the TME.

To validate these results, the expression levels of nine candidate genes encoding the proteins identified in the discovery cohort were examined in a similar cohort of 87 patients with MPM from TCGA database. The nine genes examined were MSLN, MS4A1, CD274, CD4, CD8A, CD8B, CD68, COL1A1, and COL5A1. In sarcomatoid MPM, the immune-related genes were upregulated, whereas in the epithelioid histotype, MSLN was overexpressed, confirming the intratumoral heterogeneity of MPM. Clinically, the expression of these genes according to sex and stage was consistent with the protein levels evaluated in our cohort. In addition, a strong correlation was found between the nine genes, suggesting that the expression levels of these genes could be used to classify MPM into molecular subtypes. Kaplan–Meier analysis revealed that low MSLN, COL1A1, and COL5A1 expression was significantly correlated with poor OS. Analysis of the functions and biological processes associated with the genes of interest showed that the main gene ontology biological processes involved were “collagen biosynthetic process”, “adaptative immune response”, and “lymphocyte activation”. For “molecular functions”, the strongest associations were “platelet-derived growth factor binding”, “MHC class I protein binding”, and “MHC protein binding”.

In conclusion, the present findings suggest the potential of MSLN as a biomarker for MPM diagnosis as well as a promising target for MPM immunotherapy.

## Data availability statement

The original contributions presented in the study are included in the article/**Supplementary Materials**, further inquiries can be directed to the corresponding author/s.

## Ethics statement

The Research Ethics Committee of the University of São Paulo Medical School approved this study protocol under number 600/2017. The studies were conducted in accordance with the local legislation and institutional requirements. The human samples used in this study were acquired from primarily isolated as part of your previous study for which ethical approval was obtained. Written informed consent for participation was not required from the participants or the participants’ legal guardians/next of kin in accordance with the national legislation and institutional requirements.

## Author contributions

VC: Conceptualization, Formal Analysis, Methodology, Resources, Supervision, Validation, Writing – original draft, Writing – review & editing. AQ: Data curation, Formal Analysis, Methodology, Validation, Writing – original draft. CB: Conceptualization, Data curation, Formal Analysis, Methodology, Software, Validation, Visualization, Writing – original draft. MB: Conceptualization, Data curation, Formal Analysis, Methodology, Software, Writing – original draft. AA: Conceptualization, Data curation, Formal Analysis, Methodology, Validation, Visualization, Writing – original draft. TT: Conceptualization, Data curation, Formal Analysis, Methodology, Validation, Visualization, Writing – original draft.

## Supplementary material

Supplementary Figure 1 Heatmaps showing the expression profiles of the nine analyzed genes (CD4, CD8A, CD8B, CD20, CD68, PD-L1, MSLN, COL1A1, and COL5A1) in TCGA database (Mesothelioma, Pan-Cancer Atlas) organized by histological subtype. MSLN, mesothelin; COL, collagen. The image was generated and downloaded from the UCSC Xena tool using TCGA Mesothelioma Pan-Cancer Atlas database.

Supplementary Figure 2 Mutational profile of the nine analyzed genes (CD4, CD8A, CD8B, MS4A1, CD68, CD274, MSLN, COL1A1, and COL5A1) showing immune gene- or pathway-level somatic mutations (missense mutation, amplification, or deep deletion) and their frequency in TCGA database (Mesothelioma, Pan-Cancer Atlas). MS4A1, B-lymphocyte antigen CD20; CD274, programmed cell death 1 ligand 1 (PD-L1); MSLN, mesothelin; COL, collagen. The images were downloaded from cBioPortal using TCGA Mesothelioma Pan-Cancer Atlas database.

Supplementary Figure 3 Tumor gene expression according to malignant mesothelioma tumor histology (n = 87). Boxplots showing high expression of MSLN in the epithelioid histotype compared with the biphasic type (P = 8.65E-04) and high expression of collagen type V (COL5A1) in the biphasic histotype compared with the epithelioid type (P = 1.67E-02). The boxplots show the median value, lower, and upper adjacent values, and the outside top and bottom of extreme values. The boxplots were downloaded from UALCAN and include the expression profiles for each of the genes analyzed. Gene expression comparisons according to malignant mesothelioma tumor histology were made in the platform itself using Student’s t-test. *P < 0.05; **P < 0.01. MS4A1, B-lymphocyte antigen CD20; CD274, programmed cell death 1 ligand 1 (PD-L1); MSLN, mesothelin; COL, collagen type; MESO, malignant mesothelioma.

Supplementary Figure 4 Tumor gene expression according to sex (n = 87). Boxplots show a significant correlation between high expression in transcripts per million of CD8A, CD8B, and CD274 and male patients (P = 1.79E-02, P = 2.06E-02, and P = 3.79E-04, respectively). The boxplots show the median bar value, lower and upper adjacent values, and the outside top and bottom of extreme values. The boxplots were downloaded from UALCAN including the expression profiles for each of the genes analyzed. Gene expression comparisons according to sex were made in the platform itself using Student’s t-test. *P < 0.05; **P < 0.01. MS4A1, B-lymphocyte antigen CD20; CD274, programmed cell death 1 ligand 1 (PD-L1); MSLN, mesothelin; COL, collagen type; MESO, malignant mesothelioma.

Supplementary Figure 5 Tumor gene expression according to clinical stage (n = 87). Boxplots show significantly high expression of CD4 in stage 4 compared with stage 1 (P = 3.60E-02), and significantly high expression of MSLN in stage 2 compared with stage 1 and stage 3 (P = 4.88E-02, P = 4.24E-02, respectively). The boxplots show the median bar value, lower and upper adjacent values, and the outside top and bottom of extreme values. The boxplots were downloaded from UALCAN including the expression profiles for each of the genes analyzed. Gene expression comparisons according to clinical stage were made in the platform itself using Student’s t-test. *P < 0.05; **P < 0.01. MS4A1, B-lymphocyte antigen CD20; CD274, programmed cell death 1 ligand 1 (PD-L1); MSLN, mesothelin; COL, collagen type; MESO, malignant mesothelioma.

Supplementary Figure 6 Correlation between the markers based on expression data from TCGA (Mesothelioma - Pan-Cancer Atlas) for CD4, CD8A, CD8B, MS4A1, CD68, CD274, MSLN, COL1A1, and COL5A1. Color grading represents a positive or negative correlation. The size of the dot represents Spearman’s rho; larger dots have values closer to |1| indicating a stronger correlation. *P < 0.05; **P < 0.01. The Spearman Correlation Test and the Correlation Matrix were generated by R studio software version 2022.12.0 + 353. MS4A1, B-lymphocyte antigen CD20; CD274, programmed cell death 1 ligand 1 (PD-L1); MSLN, mesothelin; COL, collagen type.

Supplementary Figure 7 Kaplan–Meier curves of the probability of survival according to follow-up time in months in patients with malignant mesothelioma from TCGA (Mesothelioma, Pan-Cancer Atlas). Curves were stratified according to (A) MSLN expression; (B) COL1A1 expression; and (C) COL5A1 expression. The blue curve represents low gene expression, and the yellow curve represents high gene expression (stratification by median expression). The image was generated and downloaded from the UCSC Xena tool using TCGA Mesothelioma - Pan-Cancer Atlas database.

Supplementary Figure 8 Cluster analysis of the protein-protein interaction network using the STRING database. The network included the nine functional genes with the highest interaction confidence score (CD4, CD8A, CD8B, MS4A1, CD68, CD274, MSLN, COL1A1, and COL5A1; P = 3.23e-10). The image was downloaded from the STRING tool after input of the nine genes.

## References

[1] Goudar RK. Review of pemetrexed in combination with cisplatin for the treatment of Malignant pleural mesothelioma. Ther Clin Risk Manage (2008) 4:205–11. doi: 10.2147/TCRM.S1603 PMC2503655 18728709

[2] Liu Z Klominek J. Regulation of matrix metalloprotease activity in Malignant mesothelioma cell lines by growth factors. Thorax (2003) 58:198–203. doi: 10.1136/thorax.58.3.198 12612292 PMC1746590

[3] Ramalingam SS Belani CP. Recent advances in the treatment of Malignant pleural mesothelioma. J Thorac Oncol (2008) 3(9):1056–64. doi: 10.1097/JTO.0b013e3181834f66 18758312

[4] Vorobiof DA Mafafo K. Malignant pleural mesothelioma: Medical treatment update. Clin Lung Cancer. (2009) 10:112–7. doi: 10.3816/CLC.2009.n.014 19362954

[5] Kulkarni NS Gupta V. Repurposing therapeutics for Malignant pleural mesothelioma (MPM)—Updates on clinical translations and future outlook. Life Sci (2022) 304:120716. doi: 10.1016/j.lfs.2022.120716 35709894

[6] Mairinger F Vollbrecht C Halbwedl I Hatz M Stacher E Gülly C. Reduced folate carrier and folylpolyglutamate synthetase, but not thymidylate synthase predict survival in pemetrexed-treated patients suffering from Malignant pleural mesothelioma. J Thorac Oncol (2013) 8(5):644–53. doi: 10.1097/JTO.0b013e318287c224 23449276

[7] Tomek S Manegold C. Chemotherapy for Malignant pleural mesothelioma: past results and recent developments. Lung Cancer. (2004) 45 Suppl 1:S103–19. doi: 10.1016/j.lungcan.2004.04.020 15261443

[8] Tomek S Emri S Krejcy K Manegold C. Chemotherapy for Malignant pleural mesothelioma: past results and recent developments. Br J Cancer. (2003) 88(2):167–74. doi: 10.1038/sj.bjc.6600673 PMC2377054 12610498

[9] Le DT Brockstedt DG Nir-Paz R Hampl J Mathur S Nemunaitis J. A live-attenuated Listeria vaccine (ANZ-100) and a live-attenuated Listeria vaccine expressing mesothelin (CRS-207) for advanced cancers: phase I studies of safety and immune induction. Clin Cancer Res (2012) 18(3):858–68. doi: 10.1158/1078-0432.CCR-11-2121 PMC3289408 22147941

[10] Krug LM Dao T Brown AB Maslak P Travis W Bekele S. WT1 peptide vaccinations induce CD4 and CD8 T cell immune responses in patients with mesothelioma and non-small cell lung cancer. Cancer Immunol Immunother. (2010) 59(10):1467–79. doi: 10.1007/s00262-010-0871-8 PMC4004362 20532500

[11] Stahel RA Weder W Felley-Bosco E Petrausch U Curioni-Fontecedro A Schmitt-Opitz I. Searching for targets for the systemic therapy of mesothelioma. Ann Oncol (2015) 26(8):1649–60. doi: 10.1093/annonc/mdv101 25722383

[12] Lanitis E Poussin M Hagemann IS Coukos G Sandaltzopoulos R Scholler N. Redirected antitumor activity of primary human lymphocytes transduced with a fully human anti-mesothelin chimeric receptor. Mol Ther (2012) 20(3):633–43. doi: 10.1038/mt.2011.256 PMC3293635 22127019

[13] Schuberth PC Hagedorn C Jensen SM Gulati P van den Broek M Mischo A. Treatment of Malignant pleural mesothelioma by fibroblast activation protein-specific re-directed T cells. J Transl Med (2013) 11:187. doi: 10.1186/1479-5876-11-187 23937772 PMC3751305

[14] Chu Q. Targeting mesothelin in solid tumours: anti-mesothelin antibody and drug conjugates. Curr Oncol Rep (2023) 25(4):309–23. doi: 10.1007/s11912-023-01367-8 36763234

[15] Grasso L Jiang Q Hassan R Nicolaides NC Kline JB. NAV-003, A bispecific antibody targeting A unique mesothelin epitope and CD3ϵ With improved cytotoxicity against humoral immunosuppressed tumors. Eur J Immunol (2023) 53(8):e2250309. doi: 10.1002/eji.202250309 37146241 PMC10524251

[16] Mairinger F Vollbrecht C Mairinger T Popper H. The issue of studies evaluating biomarkers which predict outcome after pemetrexed-based chemotherapy in Malignant pleural mesothelioma. J Thorac Oncol (2013) 8(8):e80–2. doi: 10.1097/JTO.0b013e31829b1cf9 23857409

[17] Broaddus VC Yang L Scavo LM Ernst JD Boylan AM. Asbestos induces apoptosis of human and rabbit pleural mesothelial cells *via* reactive oxygen species. J Clin Invest (1996) 98:2050–9. doi: 10.1172/JCI119010 PMC507649 8903324

[18] Carbone M Yang H. Mesothelioma: recent highlights. Ann Transl Med (2017) 5:238. doi: 10.21037/atm.2017.04.29 28706906 PMC5497108

[19] Pass HI Alimi M Carbone M Yang H Goparaju CM. Thorac Surg Clin Mesothelioma biomarkers: Discovery in search of validation. Thorac Surg Clin (2020) 30:395–423. doi: 10.1016/j.thorsurg.2020.08.001 33012429

[20] Yamaguchi N Hattori K Oh-eda M Kojima T Imai N Ochi N. A novel cytokine exhibiting megakaryocyte potentiating activity from a human pancreatic tumor cell line HPC-Y5. J Biol Chem (1994) 269(2):805–8. doi: 10.1016/S0021-9258(17)42180-6 8288629

[21] Chang K Pastan I. Molecular cloning of mesothelin, a differentiation antigen present on mesothelium, mesotheliomas, and ovarian cancers. Proc Natl Acad Sci U S A. (1996) 93(1):136–40. doi: 10.1073/pnas.93.1.136 PMC40193 8552591

[22] Hassan R Bera T Pastan I. Mesothelin: a new target for immunotherapy. Clin Cancer Res (2004) 10(12 Pt 1):3937–42. doi: 10.1158/1078-0432.CCR-03-0801 15217923

[23] Rihs HP Casjens S Raiko I Kollmeier J Lehnert M Nöfer K. Mesothelin gene variants affect soluble mesothelin-related protein levels in the plasma of asbestos-exposed males and mesothelioma patients from Germany. Biol (Basel). (2022) 11(12):1826. doi: 10.3390/biology11121826 PMC9776112 36552335

[24] Hager T Borchert S Wessolly M Mathilakathu A Mairinger E Kollmeier J. One third of Malignant pleural mesothelioma shows high immunohistochemical expression of MSLN or CXCR4 which indicates potent candidates for endo-radiotherapy. Int J Mol Sci (2023) 24(7):6356. doi: 10.3390/ijms24076356 37047331 PMC10094643

[25] Hatterer E Chauchet X Richard F Barba L Moine V Chatel L. Targeting a membrane-proximal epitope on mesothelin increases the tumoricidal activity of a bispecific antibody blocking CD47 on mesothelin-positive tumors. MAbs (2020) 12(1):1739408. doi: 10.1080/19420862.2020.1739408 32191151 PMC7153835

[26] Zhang J Qiu S Zhang Y Merino M Fetsch P Avital I. Loss of mesothelin expression by mesothelioma cells grown *in vitro* determines sensitivity to anti-mesothelin immunotoxin SS1P. Anticancer Res (2012) 32(12):5151–8. PMC6309901 23225411

[27] He X Wang L Riedel H Wang K Yang Y Dinu CZ. Mesothelin promotes epithelial-to-mesenchymal transition and tumorigenicity of human lung cancer and mesothelioma cells. Mol Cancer. (2017) 16(1):63. doi: 10.1186/s12943-017-0633-8 28288645 PMC5348784

[28] Bankhead P Loughrey MB Fernández JA Dombrowski Y McArt DG Dunne PD. QuPath: Open source software for digital pathology image analysis. Sci Rep (2017) 7(1):16878. doi: 10.1038/s41598-017-17204-5 29203879 PMC5715110

[29] Chandrashekar DS Karthikeyan SK Korla PK Patel H Shovon AR Athar M. UALCAN: An update to the integrated cancer data analysis platform. Neoplasia (2022) 25:18–27. doi: 10.1016/j.neo.2022.01.001 35078134 PMC8788199

[30] Chandrashekar DS Bashel B Balasubramanya SAH Creighton CJ Ponce-Rodriguez I Chakravarthi BVSK. UALCAN: A portal for facilitating tumor subgroup gene expression and survival analyses. Neoplasia (2017) 19(8):649–58. doi: 10.1016/j.neo.2017.05.002 PMC5516091 28732212

[31] Cerami E Gao J Dogrusoz U Gross BE Sumer SO Aksoy BA. The cBio cancer genomics portal: an open platform for exploring multidimensional cancer genomics data. Cancer Discovery (2012) 2(5):401–4. doi: 10.1158/2159-8290.CD-12-0095 PMC3956037 22588877

[32] Gao J Aksoy BA Dogrusoz U Dresdner G Gross B Sumer SO. Integrative analysis of complex cancer genomics and clinical profiles using the cBioPortal. Sci Signal (2013) 6(269):pl1. doi: 10.1126/scisignal.2004088 23550210 PMC4160307

[33] Goldman MJ Craft B Hastie M Repečka K McDade F Kamath A. Visualizing and interpreting cancer genomics data *via* the Xena platform. Nat Biotechnol (2020) 38(6):675–8. doi: 10.1038/s41587-020-0546-8 PMC7386072 32444850

[34] Szklarczyk D Gable AL Nastou KC Lyon D Kirsch R Pyysalo S. The STRING database in 2021: customizable protein-protein networks, and functional characterization of user-uploaded gene/measurement sets. Nucleic Acids Res (2021) 49(D1):D605–12. doi: 10.1093/nar/gkaa1074 PMC7779004 33237311

[35] Snel B Lehmann G Bork P Huynen MA. STRING: a web-server to retrieve and display the repeatedly occurring neighbourhood of a gene. Nucleic Acids Res (2000) 28(18):3442–4. doi: 10.1093/nar/28.18.3442 PMC110752 10982861

[36] Bretagnolle J Huber-Carol C. Effects of omitting covariates in Cox’s model for survival data. Scandinavian J Stat (1988) 15(2):125–38.

[37] Lacourt A Gramond C Rolland P Ducamp S Audignon S Astoul P. Occupational and non-occupational attributable risk of asbestos exposure for Malignant pleural mesothelioma. Thorax (2014) 69(6):532–9. doi: 10.1136/thoraxjnl-2013-203744 24508707

[38] Bibby AC Tsim S Kanellakis N Ball H Talbot DC Blyth KG. Malignant pleural mesothelioma: an update on investigation, diagnosis and treatment. Eur Respir Rev (2016) 25(142):472–86. doi: 10.1183/16000617.0063-2016 PMC9487555 27903668

[39] Kondola S Manners D Nowak AK. Malignant pleural mesothelioma: an update on diagnosis and treatment options. Ther Adv Respir Dis (2016) 10(3):275–88. doi: 10.1177/1753465816628800 PMC5933604 26873306

[40] Borgeaud M Kim F Friedlaender A Lococo F Addeo A Minervini F. The evolving role of immune-checkpoint inhibitors in Malignant pleural mesothelioma. J Clin Med (2023) 12(5):1757. doi: 10.3390/jcm12051757 36902544 PMC10003250

[41] Baas P Scherpereel A Nowak AK Fujimoto N Peters S Tsao AS. First-line nivolumab plus ipilimumab in unresectable Malignant pleural mesothelioma (CheckMate 743): a multicentre, randomised, open-label, phase 3 trial. Lancet (2001) 397(10272):375–86. doi: 10.1016/S0140-6736(20)32714-8 33485464

[42] Keytruda® (pembrolizumab) plus chemotherapy significantly improved overall survival versus chemotherapy alone as first-line treatment for advanced Malignant pleural mesothelioma. News release. Merck . Available at: https://clinicaltrials.gov/ct2/show/NCT02784171 (Accessed March 10, 2023).

[43] Tomasetti M Monaco F Strogovets O Volpini L Valentino M Amati M. ATG5 as biomarker for early detection of Malignant mesothelioma. BMC Res Notes. (2023) 16(1):61. doi: 10.1186/s13104-023-06330-1 37095543 PMC10127310

[44] Tian L Zeng R Wang X Shen C Lai Y Wang M. Prognostic significance of soluble mesothelin in Malignant pleural mesothelioma: a meta-analysis. Oncotarget (2017) 8(28):46425–35. doi: 10.18632/oncotarget.17436 PMC5542278 28507279

[45] Ledda C Senia P Rapisarda V. Biomarkers for early diagnosis and prognosis of Malignant pleural mesothelioma: the quest goes on. Cancers (Basel). (2018) 10(6):203. doi: 10.3390/cancers10060203 29914087 PMC6025035

[46] Borea F Franczak MA Garcia M Perrino M Cordua N Smolenski RT. Target therapy in Malignant pleural mesothelioma: hope or mirage? Int J Mol Sci (2023) 24(11):9165. doi: 10.3390/ijms24119165 37298116 PMC10253134

[47] Hassan R Bullock S Premkumar A Kreitman RJ Kindler H Willingham MC. Phase I study of SS1P, a recombinant anti-mesothelin immunotoxin given as a bolus I.V. infusion to patients with mesothelin-expressing mesothelioma, ovarian, and pancreatic cancers. Clin Cancer Res (2007) 13(17):5144–9. doi: 10.1158/1078-0432.CCR-07-0869 17785569

[48] Kreitman RJ Hassan R Fitzgerald DJ Pastan I. Phase I trial of continuous infusion anti-mesothelin recombinant immunotoxin SS1P. Clin Cancer Res (2009) 15(16):5274–9. doi: 10.1158/1078-0432.CCR-09-0062 PMC2754261 19671873

[49] Hassan R Miller AC Sharon E Thomas A Reynolds JC Ling A. Major cancer regressions in mesothelioma after treatment with an anti-mesothelin immunotoxin and immune suppression. Sci Transl Med (2013) 5(208):208ra147. doi: 10.1126/scitranslmed.3006941 PMC6369691 24154601

[50] Hassan R Alewine C Mian I Spreafico A Siu LL Gomez-Roca C. Phase 1 study of the immunotoxin LMB-100 in patients with mesothelioma and other solid tumors expressing mesothelin. Cancer (2020) 126(22):4936–47. doi: 10.1002/cncr.33145 PMC8552963 32870522

[51] Bharadwaj U Marin-Muller C Li M Chen C Yao Q. Mesothelin confers pancreatic cancer cell resistance to TNF-α-induced apoptosis through Akt/PI3K/NF-κB activation and IL-6/Mcl-1 overexpression. Mol Cancer. (2011) 10:106. doi: 10.1186/1476-4598-10-106 21880146 PMC3175472

[52] Weidemann S Gagelmann P Gorbokon N Lennartz M Menz A Luebke AM. Mesothelin expression in human tumors: A tissue microarray study on 12,679 tumors. Biomedicines (2021) 9(4):397. doi: 10.3390/biomedicines9040397 33917081 PMC8067734

[53] Thomas AM Santarsiero LM Lutz ER Armstrong TD Chen YC Huang LQ. Mesothelin-specific CD8(+) T cell responses provide evidence of *in vivo* cross-priming by antigen-presenting cells in vaccinated pancreatic cancer patients. J Exp Med (2004) 200(3):297–306. doi: 10.1084/jem.20031435 15289501 PMC2211979

[54] Ho M Hassan R Zhang J Wang QC Onda M Bera T. Humoral immune response to mesothelin in mesothelioma and ovarian cancer patients. Clin Cancer Res (2005) 11(10):3814–20. doi: 10.1158/1078-0432.CCR-04-2304 15897581

[55] Carmona R Cano E Grueso E Ruiz-Villalba A Bera TK Gaztambide J. Peritoneal repairing cells: a type of bone marrow derived progenitor cells involved in mesothelial regeneration. J Cell Mol Med (2011) 15(5):1200–9. doi: 10.1111/j.1582-4934.2010.01087.x PMC3822632 20477904

