# Supplementary figures and images for: Mesothelin expression remodeled the immune-matrix tumor microenvironment predicting the risk of death in patients with malignant pleural mesothelioma
Source: Front Immunol. 2023 Oct 12;14:1268927. doi: 10.3389/fimmu.2023.1268927 (PMC10601658; doi:10.3389/fimmu.2023.1268927)

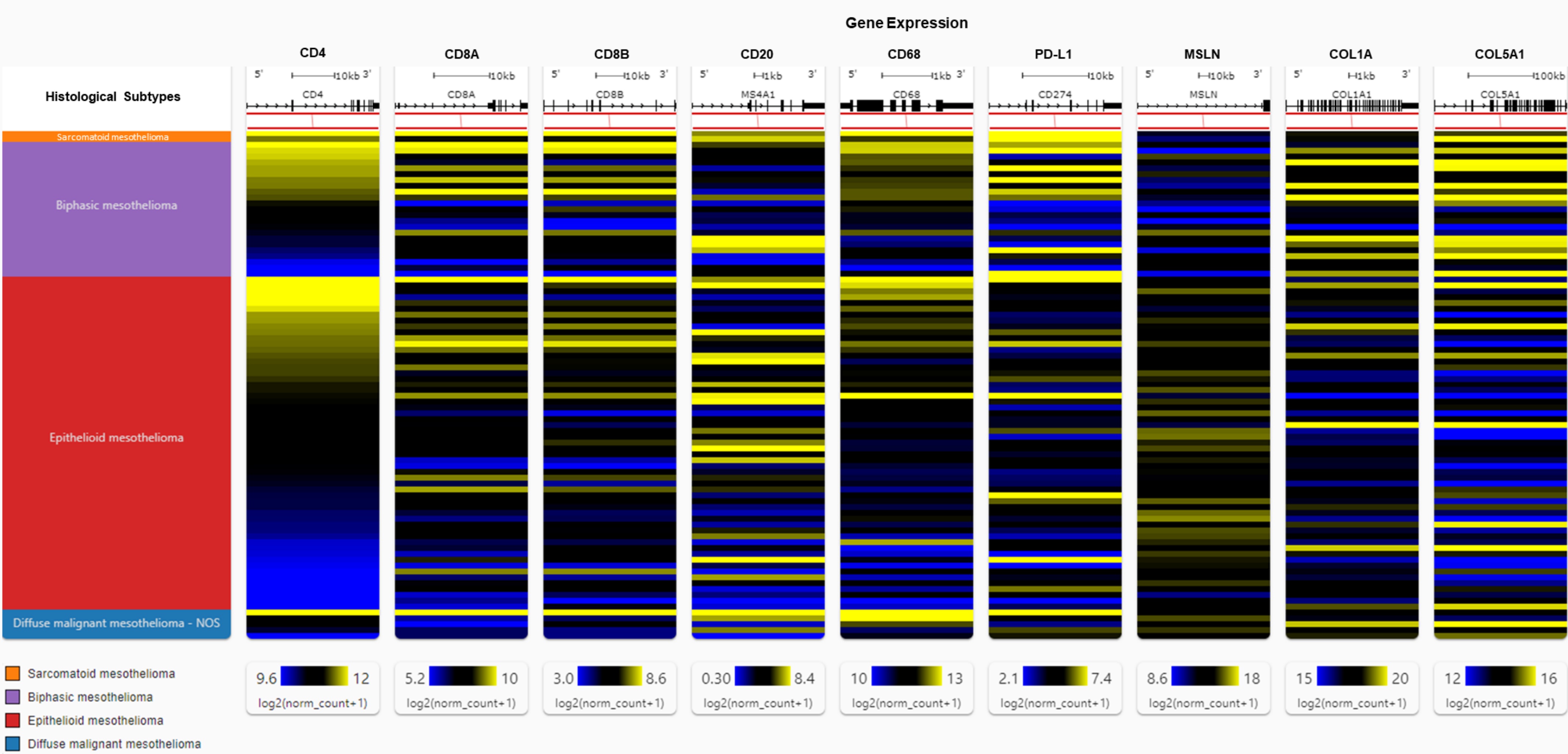

Supplement: Supplementary Figure 1 — Heatmaps showing the expression profiles of the nine analyzed genes (CD4, CD8A, CD8B, CD20, CD68, PD-L1, MSLN, COL1A1, and COL5A1) in TCGA database (Mesothelioma, Pan-Cancer Atlas) organized by histological subtype. MSLN, mesothelin; COL, collagen. The image was generated and downloaded from the UCSC Xena tool using TCGA Mesothelioma Pan-Cancer Atlas database. [file Image_1.jpeg]

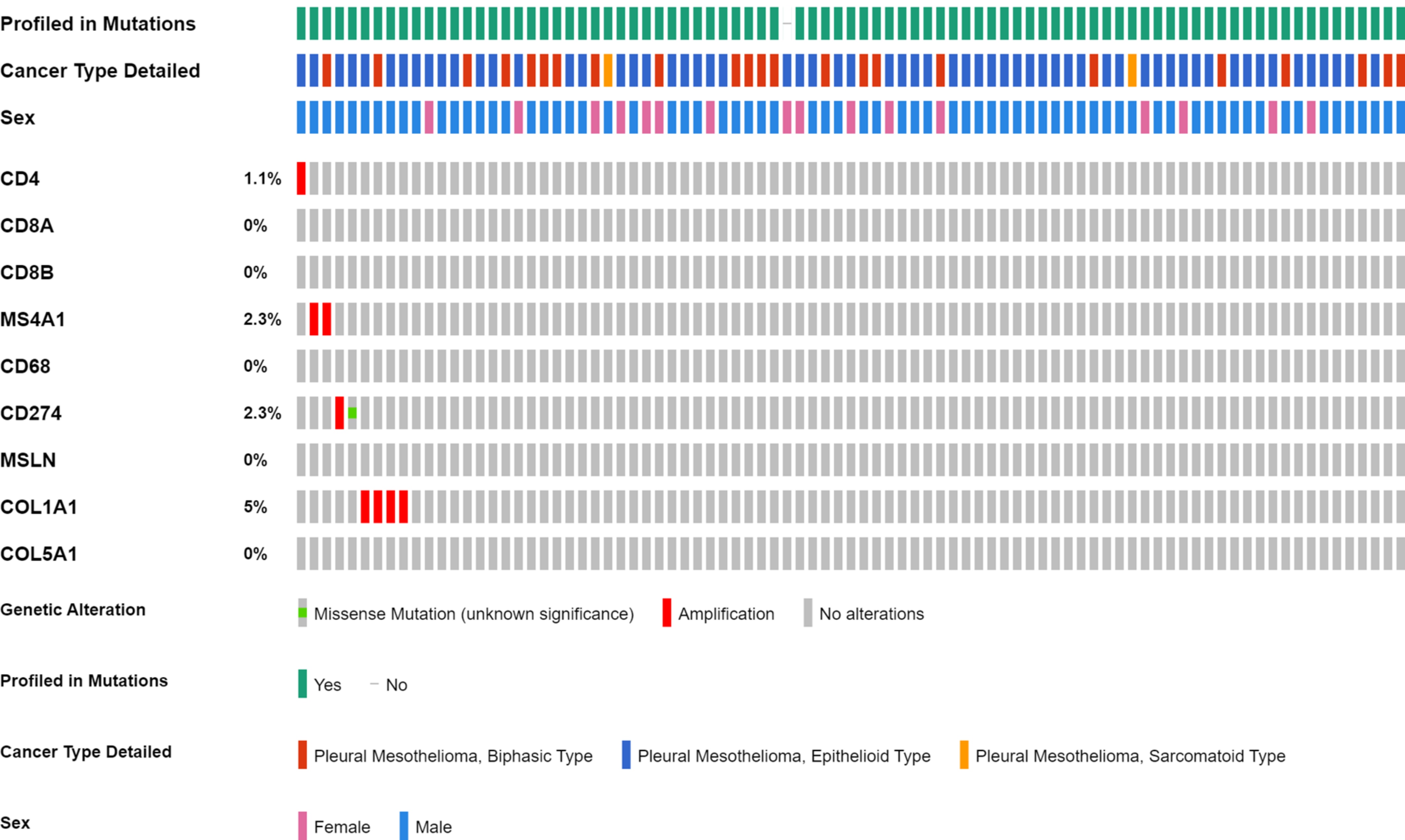

Supplement: Supplementary Figure 2 — Mutational profile of the nine analyzed genes (CD4, CD8A, CD8B, MS4A1, CD68, CD274, MSLN, COL1A1, and COL5A1) showing immune gene- or pathway-level somatic mutations (missense mutation, amplification, or deep deletion) and their frequency in TCGA database (Mesothelioma, Pan-Cancer Atlas). MS4A1, B-lymphocyte antigen CD20; CD274, programmed cell death 1 ligand 1 (PD-L1); MSLN, mesothelin; COL, collagen. The images were downloaded from cBioPortal using TCGA Mesothelioma Pan-Cancer Atlas database. [file Image_2.jpeg]

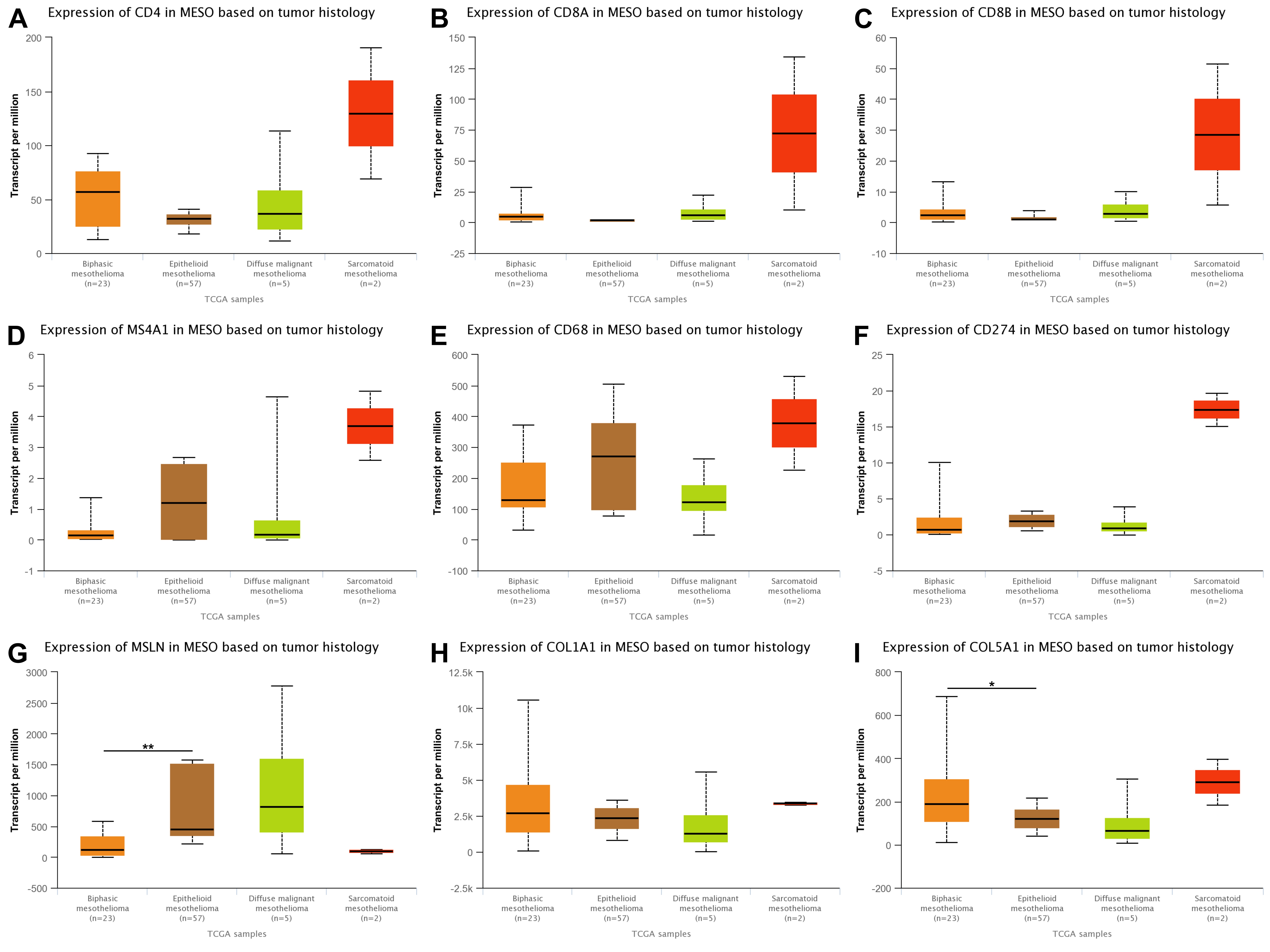

Supplement: Supplementary Figure 3 — Tumor gene expression according to malignant mesothelioma tumor histology (n = 87). Boxplots showing high expression of MSLN in the epithelioid histotype compared with the biphasic type (P = 8.65E-04) and high expression of collagen type V (COL5A1) in the biphasic histotype compared with the epithelioid type (P = 1.67E-02). The boxplots show the median value, lower, and upper adjacent values, and the outside top and bottom of extreme values. The boxplots were downloaded from UALCAN and include the expression profiles for each of the genes analyzed. Gene expression comparisons according to malignant mesothelioma tumor histology were made in the platform itself using Student’s t-test. *P < 0.05; **P < 0.01. MS4A1, B-lymphocyte antigen CD20; CD274, programmed cell death 1 ligand 1 (PD-L1); MSLN, mesothelin; COL, collagen type; MESO, malignant mesothelioma. [file Image_3.jpeg]

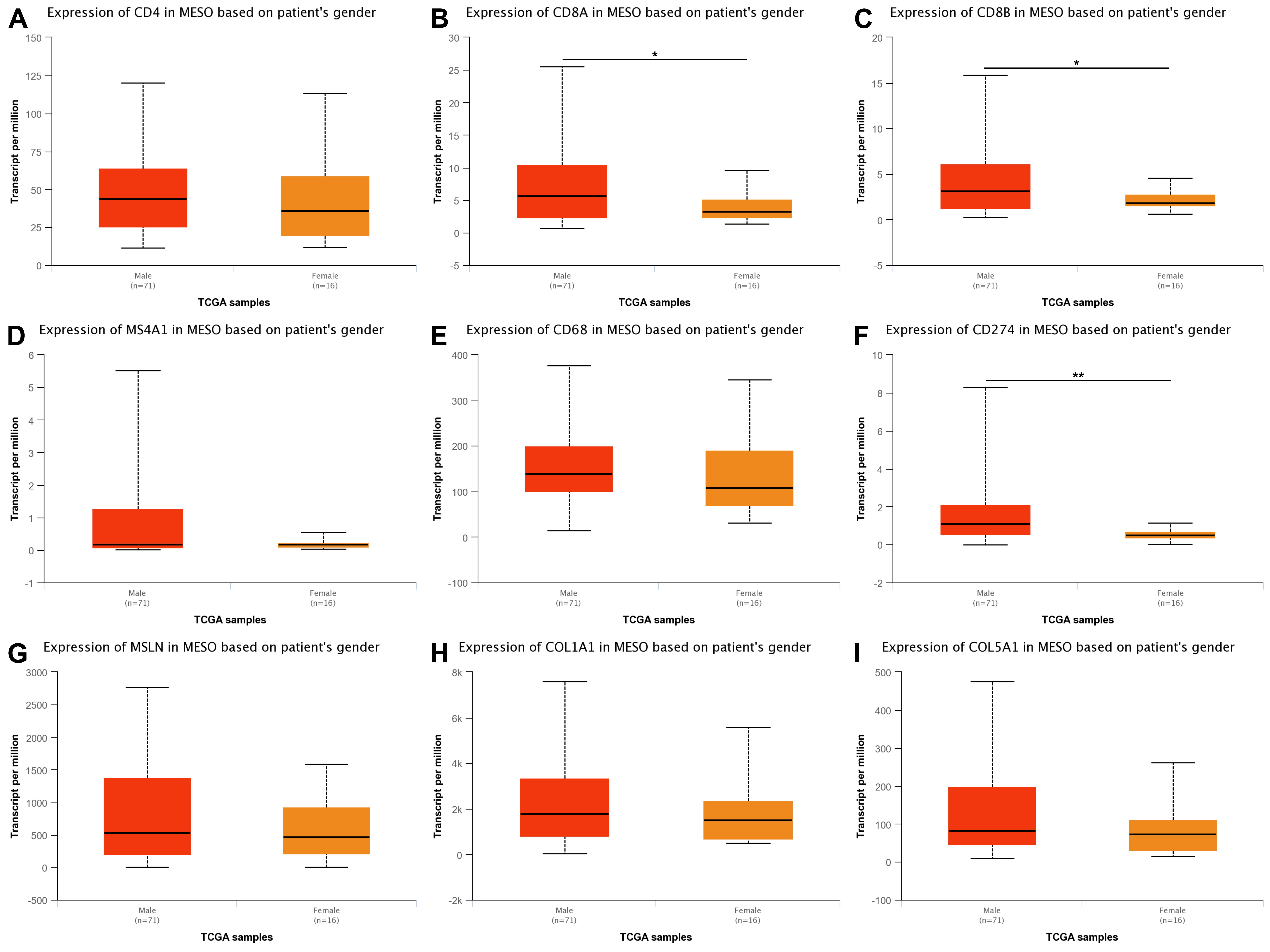

Supplement: Supplementary Figure 4 — Tumor gene expression according to sex (n = 87). Boxplots show a significant correlation between high expression in transcripts per million of CD8A, CD8B, and CD274 and male patients (P = 1.79E-02, P = 2.06E-02, and P = 3.79E-04, respectively). The boxplots show the median bar value, lower and upper adjacent values, and the outside top and bottom of extreme values. The boxplots were downloaded from UALCAN including the expression profiles for each of the genes analyzed. Gene expression comparisons according to sex were made in the platform itself using Student’s t-test. *P < 0.05; **P < 0.01. MS4A1, B-lymphocyte antigen CD20; CD274, programmed cell death 1 ligand 1 (PD-L1); MSLN, mesothelin; COL, collagen type; MESO, malignant mesothelioma. [file Image_4.jpeg]

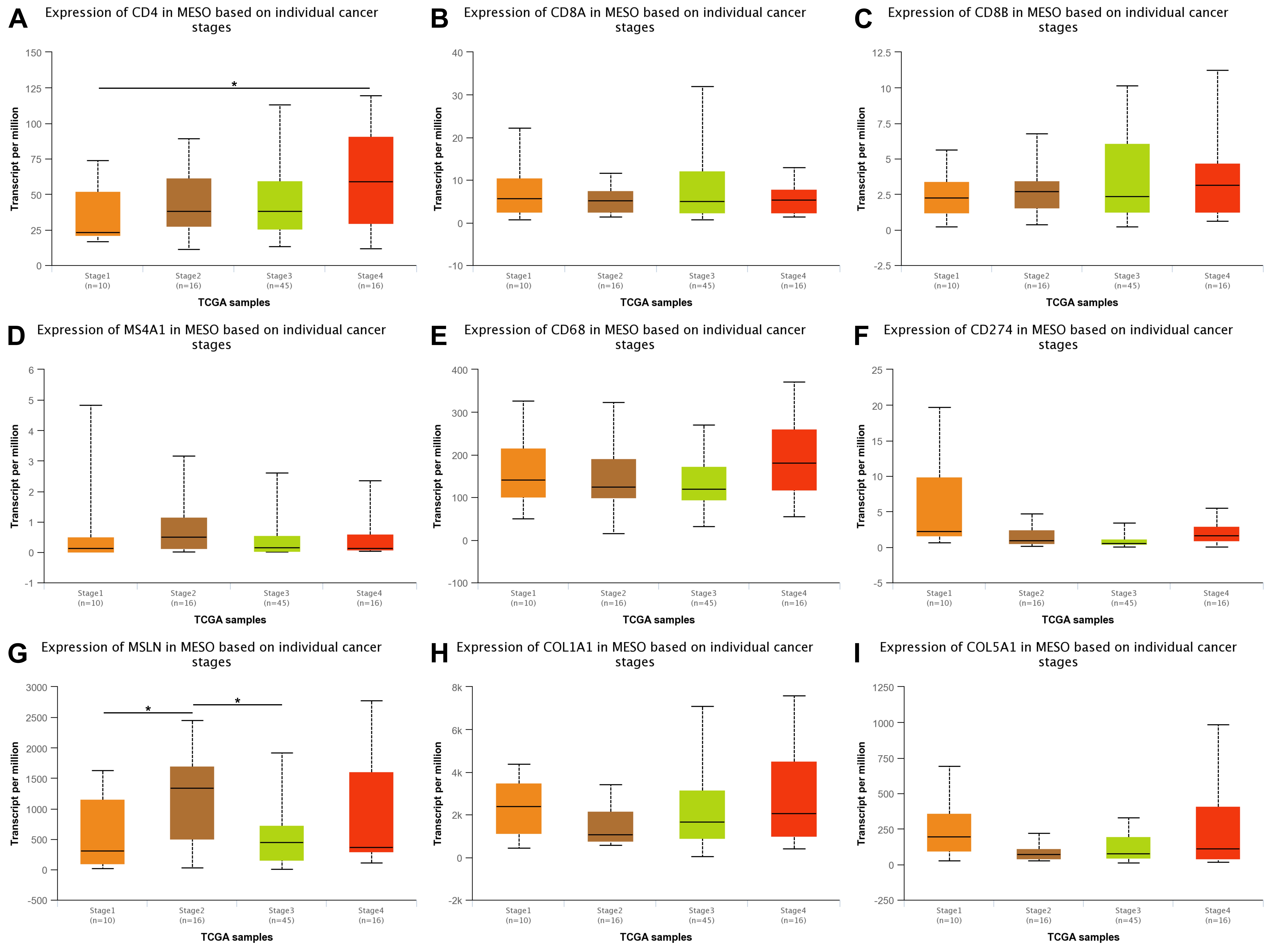

Supplement: Supplementary Figure 5 — Tumor gene expression according to clinical stage (n = 87). Boxplots show significantly high expression of CD4 in stage 4 compared with stage 1 (P = 3.60E-02), and significantly high expression of MSLN in stage 2 compared with stage 1 and stage 3 (P = 4.88E-02, P = 4.24E-02, respectively). The boxplots show the median bar value, lower and upper adjacent values, and the outside top and bottom of extreme values. The boxplots were downloaded from UALCAN including the expression profiles for each of the genes analyzed. Gene expression comparisons according to clinical stage were made in the platform itself using Student’s t-test. *P < 0.05; **P < 0.01. MS4A1, B-lymphocyte antigen CD20; CD274, programmed cell death 1 ligand 1 (PD-L1); MSLN, mesothelin; COL, collagen type; MESO, malignant mesothelioma. [file Image_5.jpeg]

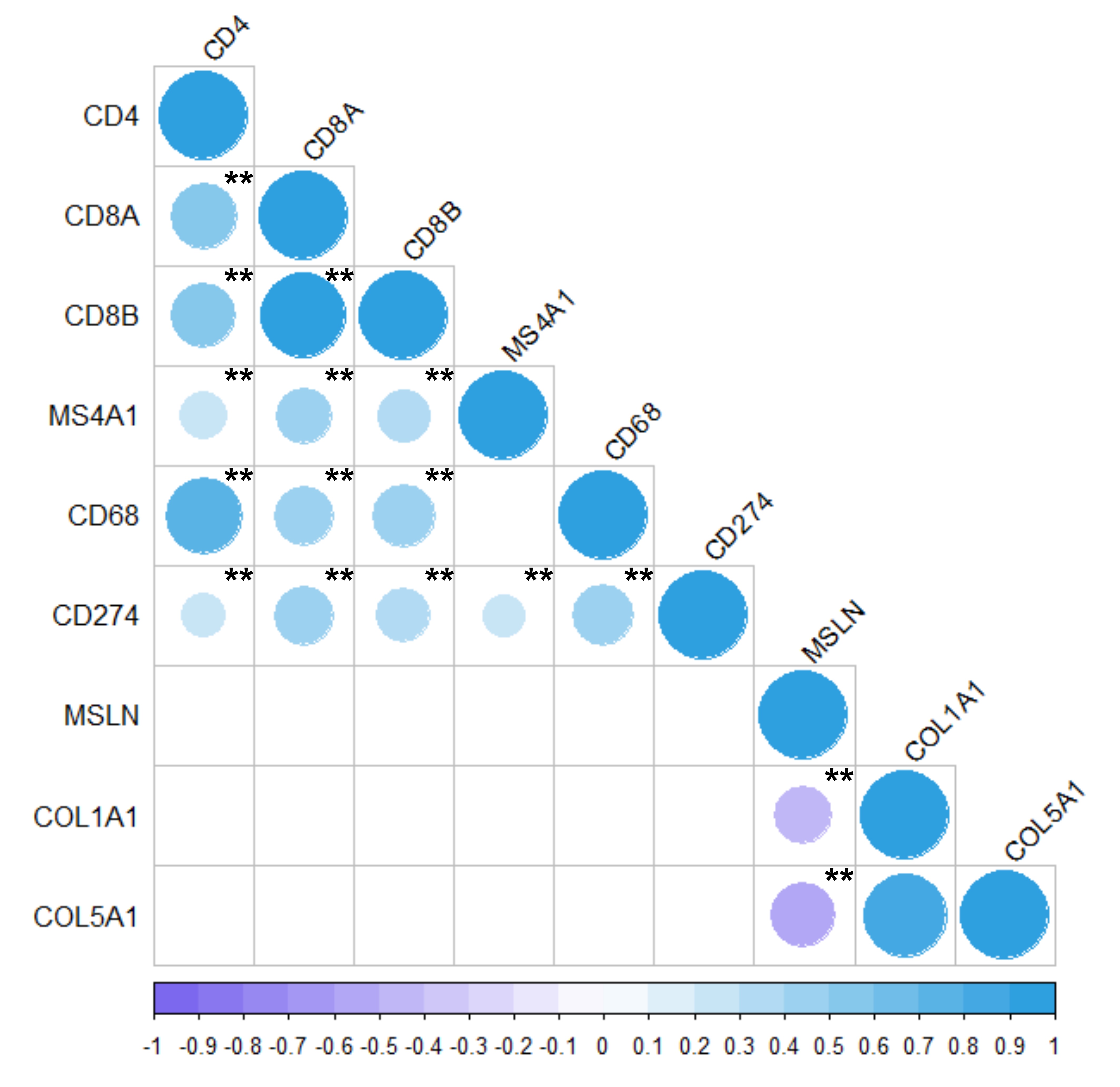

Supplement: Supplementary Figure 6 — Correlation between the markers based on expression data from TCGA (Mesothelioma - Pan-Cancer Atlas) for CD4, CD8A, CD8B, MS4A1, CD68, CD274, MSLN, COL1A1, and COL5A1. Color grading represents a positive or negative correlation. The size of the dot represents Spearman’s rho; larger dots have values closer to |1| indicating a stronger correlation. *P < 0.05; **P < 0.01. The Spearman Correlation Test and the Correlation Matrix were generated by R studio software version 2022.12.0 + 353. MS4A1, B-lymphocyte antigen CD20; CD274, programmed cell death 1 ligand 1 (PD-L1); MSLN, mesothelin; COL, collagen type. [file Image_6.jpeg]

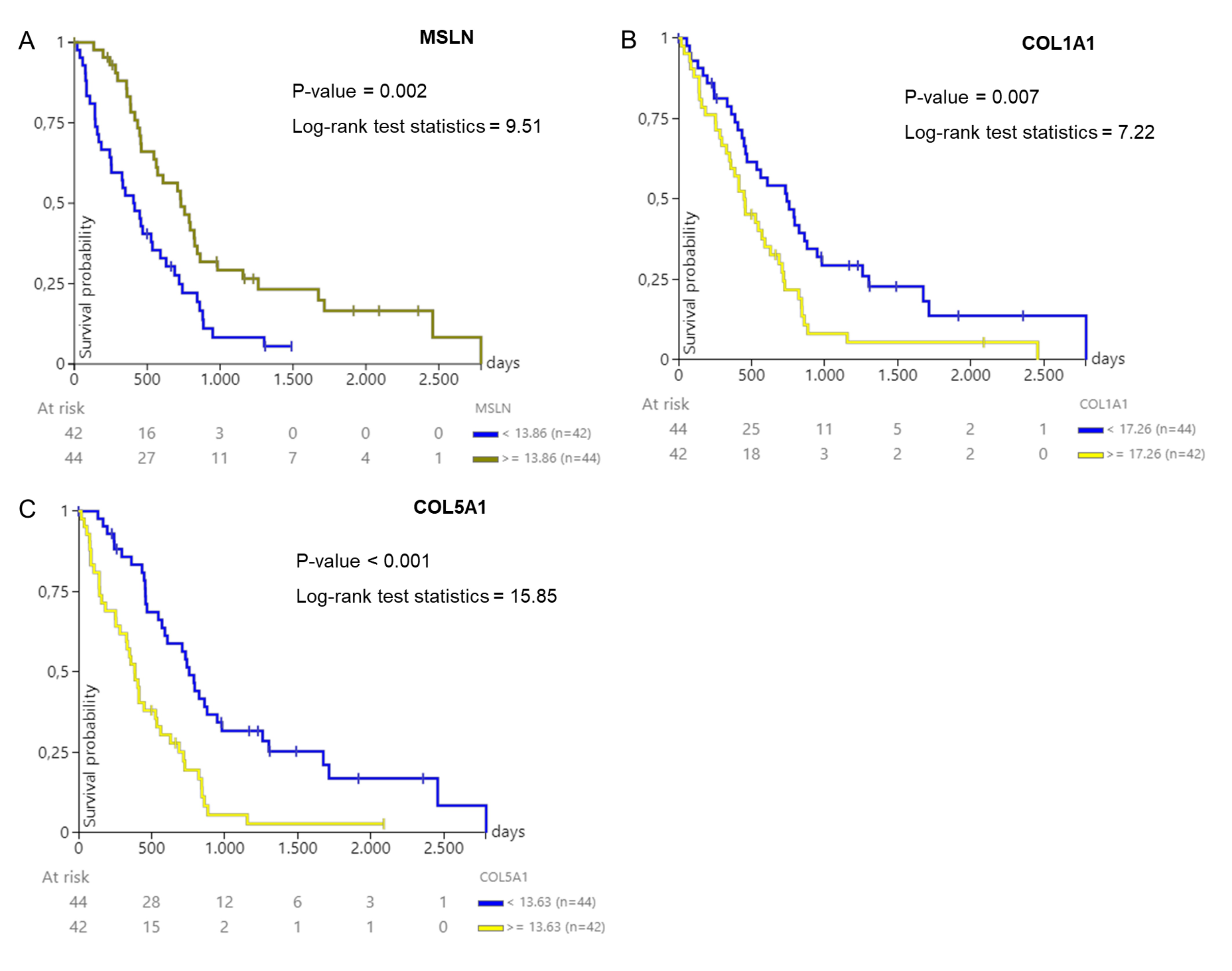

Supplement: Supplementary Figure 7 — Kaplan–Meier curves of the probability of survival according to follow-up time in months in patients with malignant mesothelioma from TCGA (Mesothelioma, Pan-Cancer Atlas). Curves were stratified according to (A) MSLN expression; (B) COL1A1 expression; and (C) COL5A1 expression. The blue curve represents low gene expression, and the yellow curve represents high gene expression (stratification by median expression). The image was generated and downloaded from the UCSC Xena tool using TCGA Mesothelioma - Pan-Cancer Atlas database. [file Image_7.jpeg]

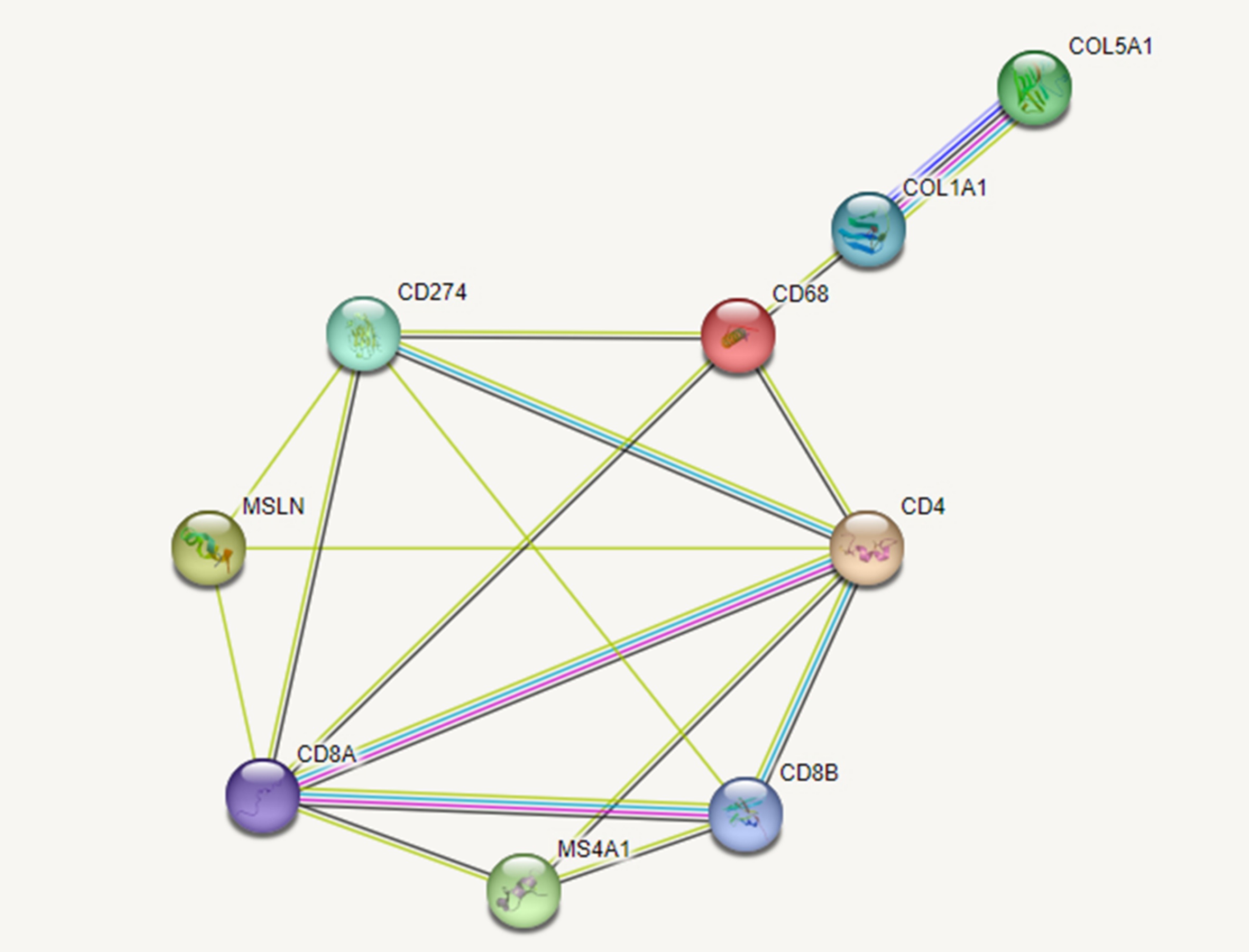

Supplement: Supplementary Figure 8 — Cluster analysis of the protein-protein interaction network using the STRING database. The network included the nine functional genes with the highest interaction confidence score (CD4, CD8A, CD8B, MS4A1, CD68, CD274, MSLN, COL1A1, and COL5A1; P = 3.23e-10). The image was downloaded from the STRING tool after input of the nine genes. [file Image_8.jpeg]
