# Supplementary material for: Mesothelin expression remodeled the immune-matrix tumor microenvironment predicting the risk of death in patients with malignant pleural mesothelioma
Source: Front Immunol. 2023 Oct 12;14:1268927. doi: 10.3389/fimmu.2023.1268927 (PMC10601658; doi:10.3389/fimmu.2023.1268927)
Supplement: Supplementary file 9 [file Table_1.docx]

**Supp. Table 1.** Functional enrichment of correlated genes

| **Gene Ontology** | | | |  |
| --- | --- | --- | --- | --- |
| ***Biological Process*** |  | |  |  |
| **Description** | **GO-term** | **Strength** | | **FDR** |
| Collagen biosynthetic process | GO:0032964 | 2.68 | | 0.0269 |
| Adaptive immune response | GO:0002250 | 1.44 | | 0.0269 |
| Lymphocyte activation | GO:0046649 | 1.35 | | 0.0345 |
| Regulation of immune response | GO:0050776 | 1.16 | | 0.0068 |
| Cell activation | GO:0001775 | 1.08 | | 0.0087 |
| Leukocyte activation | GO:0045321 | 1.07 | | 0.0375 |
| Regulation of immune system process | GO:0002682 | 1.00 | | 0.0068 |
| Immune response | GO:0006955 | 0.91 | | 0.0345 |
| Immune system process | GO:0002376 | 0.79 | | 0.0324 |
| ***Molecular Function*** |  |  | |  |
| **Description** | **GO-term** | **Strength** | | **FDR** |
| Platelet-derived growth factor binding | GO:0048407 | 2.60 | | 0.0158 |
| MHC class I protein binding | GO:0042288 | 2.34 | | 0.0280 |
| MHC protein binding | GO:0042287 | 2.30 | | 0.0015 |
| Immunoglobulin binding | GO:0019865 | 2.28 | | 0.0302 |
| MHC protein complex binding | GO:0023023 | 2.24 | | 0.0303 |
| Extracellular matrix structural constituent conferring tensile strength | GO:0030020 | 2.19 | | 0.0329 |
| Coreceptor activity | GO:0015026 | 2.14 | | 0.0021 |
| Extracellular matrix structural constituent | GO:0005201 | 1.74 | | 0.0158 |
| **KEGG Pathway** | | | |  |
| **Description** | **Pathway** | **Strength** | | **FDR** |
| Primary immunodeficiency | hsa05340 | 2.25 | | 7.37e-05 |
| Antigen processing and presentation | hsa04612 | 2.02 | | 0.00025 |
| Hematopoietic cell lineage | hsa04640 | 1.98 | | 2.16e-05 |
| T cell receptor signaling pathway | hsa04660 | 1.81 | | 0.00080 |
| Cell adhesion molecules | hsa04514 | 1.80 | | 5.31e-05 |
| Yersinia infection | hsa05135 | 1.72 | | 0.0012 |
| PD-L1 expression and PD-1 checkpoint pathway in cancer | hsa05235 | 1.69 | | 0.0354 |
| Protein digestion and absorption | hsa04974 | 1.64 | | 0.0397 |
| **Reactome Pathway** | | | |  |
| **Description** | **Pathway** | **Strength** | | **FDR** |
| PD-1 signaling | HSA-389948 | 2.36 | | 0.0284 |
| Nef-mediates down modulation of cell surface receptors by recruiting   them to clathrin adapters | HSA-164938 | 2.32 | | 0.0284 |
| Syndecan interactions | HSA-3000170 | 2.21 | | 0.0284 |
| MET activates PTK2 signaling | HSA-8874081 | 2.16 | | 0.0284 |
| Collagen chain trimerization | HSA-8948216 | 1.99 | | 0.0418 |
| Immunoregulatory interactions between a Lymphoid and a  non-Lymphoid cell | HSA-198933 | 1.70 | | 0.0265 |
| Adaptive Immune System | HSA-1280218 | 1.17 | | 0.0194 |
| Immune System | HSA-168256 | 0.82 | | 0.0284 |

**Abbreviations:** FDR, false discovery rate.
